# A biradical oxo-molybdenum complex containing semiquinone and *o*-aminophenol benzoxazole-based ligands†

**DOI:** 10.1039/d0ra06351g

**Published:** 2020-11-09

**Authors:** Mina Nasibipour, Elham Safaei, Andrzej Wojtczak, Zvonko Jagličić, Agustín Galindo, Marzieh Sadat Masoumpour

**Affiliations:** Department of Chemistry, College of Sciences, Shiraz University 71454 Shiraz Iran e.safaei@shirazu.ac.ir; Nicolaus Copernicus University, Faculty of Chemistry 87-100 Torun Poland; Institute of Mathematics, Physics and Mechanics & Faculty of Civil and Geodetic Engineering, University of Ljubljana Jadranska 19 Ljubljana Slovenia; Departamento de Química Inorgánica, Facultad de Química, Universidad de Sevilla Aptdo. 1203 41071 Sevilla Spain; Department of Chemistry, Estahban Higher Education Center Estahban 74519-44655 Iran

## Abstract

We report a new mononuclear molybdenum(iv) complex, MoOL^BIS^L^SQ^, in which L^SQ^ (2,4-di-*tert*-butyl *o*-semibenzoquinone ligand) has been prepared from the reaction of the *o*-iminosemibenzoquinone form of a tridentate non-innocent benzoxazole ligand, L^BIS^, and MoO_2_(acac)_2_. The complex was characterized by X-ray crystallography, elemental analysis, IR and UV-vis spectroscopy and magnetic susceptibility measurements. The crystal structure of MoOL^BIS^L^SQ^ revealed a distorted octahedral geometry around the metal centre, surrounded by one O and two N atoms of L^BIS^ and two O atoms of L^SQ^. The effective magnetic moment (*μ*_eff_) of MoOL^BIS^L^SQ^ decreased from 2.36 to 0.2 μ_B_ in the temperature range of 290 to 2 K, indicating a singlet ground state caused by antiferromagnetic coupling between the metal and ligand centred unpaired electrons. Also, the latter led to the EPR silence of the complex. Cyclic voltammetry (CV) studies indicate both ligand and metal-centered redox processes. MoOL^BIS^L^SQ^ was applied as a catalyst for the oxidative cleavage of cyclohexene to adipic acid and selective oxidation of sulfides to sulfones with aqueous hydrogen peroxide.

## Abstract 2

Biradical molybdenum(iv) complex, MoOL^BIS^L^SQ^, has been prepared from the reaction of the *o*-iminosemibenzoquinone form of a tridentate non-innocent benzoxazole ligand, L^BIS^, and MoO_2_(acac)_2_ and used as catalyst in oxidation reaction..

## Graphical abstract

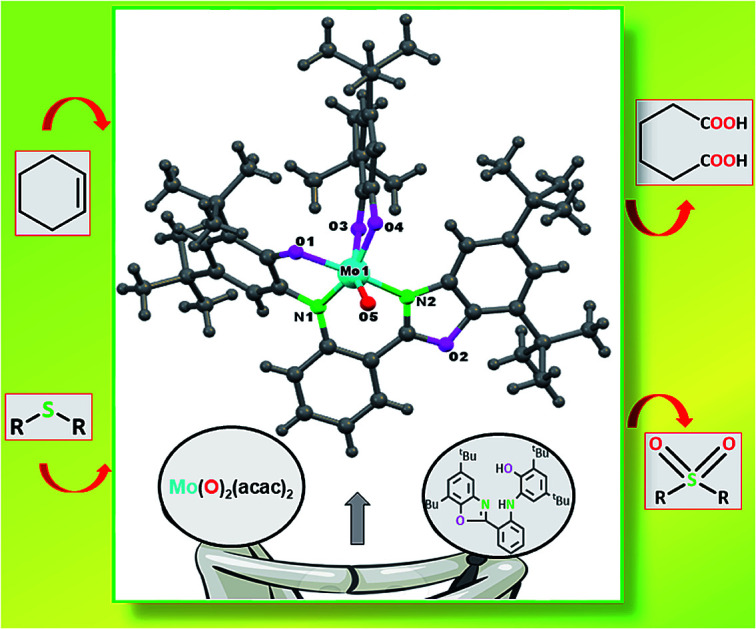

## Introduction

Scientists have discovered in recent decades that some enzymes with special ligands are able to perform redox processes at both their metal centers and their coordinated ligands. The variation of oxidation states of such ligands is a special and important feature that causes their bound metals to change their oxidation states. These are known as non-innocent ligands. The term (redox) “non-innocent ligand” is used in the scientific literature to reflect the unclear oxidation state of a ligand in a metal complex. This characteristic leads to obscurity in the exact defined oxidation state of the bound metal center. Hence complexes containing non-innocent ligands are known as redox active metal complexes. This feature of the ligands is important for the catalytic activity of the broadly investigated complexes in which redox reactions could be ligand or metal localized. In this way, metal complexes of non-innocent ligands are now undergoing a renaissance in synthetic chemistry because they are good structural and functional models for the mentioned enzymes.^1^

Molybdenum is used in some groups of oxotransferases or hydrolases enzymes, in which, they catalyze important and vital reactions like oxygen atom transfer reactions (OAT) and water transfer reactions to substrates, respectively. These enzymes, based on their oxidized active center structure are divided into three different categories referred to sulfite and xanthine oxidase and DMSO reductase. All these enzymes contain a mononuclear molybdenum-oxo center.^2^

Due to the importance of these reactions the active sites of oxotransferases have been structurally mimicked by a large number of molybdenum complexes.^3^ They catalyze a variety of reactions, especially oxidation of several organic substrates with various oxidants, such as *tert*-butyl hydroperoxide (TBHP) and H_2_O_2_.^4^ It is worth mentioning that during the last decades, scientists paid attention to H_2_O_2_ as a clean oxidant and appropriate alternative to the environmentally hazardous ones for metal-catalyzed oxidation of the organic substrate.^5^ However, use these complexes is sometimes affected by problems like a small rate of active oxygen preparation and easy decomposition. Therefore, scientists have focused on effective catalysts that can activate hydrogen peroxide without any decomposition. Complexes with V, VI, and VII group transition metals, especially molybdenum, are one of the best candidates to prevail in these limitations.

**Scheme 1:**
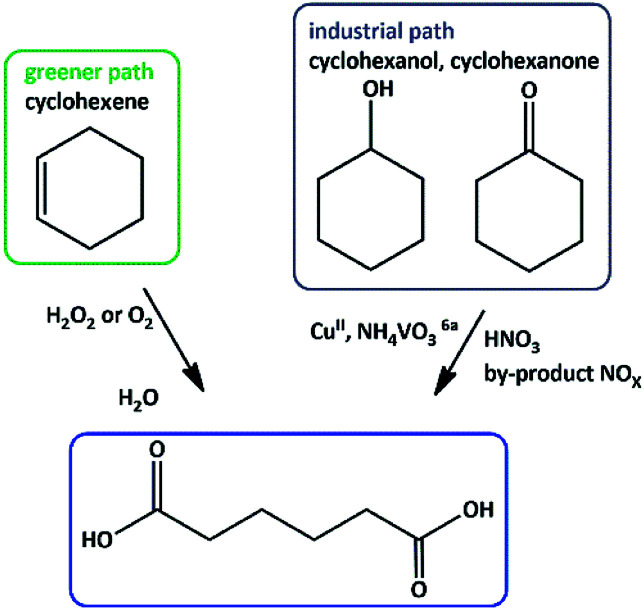
Proposed routes for the synthesis of adipic acid (AA).^9^

Adipic acid (AA) or 1,6-hexanedioic acid, is an essential raw compound in chemical industries, such as nylon 6 production. Oxidation of KA (ketone–alcohol) oil (cyclohexanone–cyclohexanol) mixture by nitric acid is the current industrial way to produce adipic acid.^6^ Generating of a large amount of nitrous oxide (N_2_O), *i.e.* environmentally harmful gas, is the major defect of this process.^7^ One-step production of adipic acid *via* oxidative cleavage of cyclohexene using H_2_O_2_ has been presented as a green alternative method (Scheme 1).^8^

It is worth saying that in recent years a rapid AA production *via* high temperatures and pressure,^10^*via* membrane reactors,^11^ and microwave settings^12^ has been described.

On the other hand, there is air pollution and acid rain because of the sulfur pollutants in fuels as an anthropogenic source of atmospheric SO_*x*_. Therefore, to decrease the sulfur oxide emissions, the hydrodesulfurization of petroleum on molybdenum-containing catalysts in high temperature and high H_2_ pressure conditions has long been one of the major catalytic methods used in the industry.^13^

Only a few sulfur compounds can be unchanged in this process and have slow desulfurization with excessive conditions what makes the process expensive. Especially, some fused-ring thiophenes, *e.g.* dibenzothiophene (DBT) are challenging compounds.

Following the idea that the redox transformations could be modulated by the *o*-aminophenol as an “electron reservoir” ligand,^1*c*,*d*^ our present research is committed to the development of the Mo complex containing redox-active ligand.

In the paper, we describe the preparation and characterization of oxo molybdenum complex coordinated by the redox-active *o*-amino phenol ligand providing N,O additional donors capable of two and tridentate coordination and apply this complex for AA production under mild and safe conditions (low temperature, eluding the H_2_O_2_ decomposition, one-step reaction, cheap catalyst, and atmospheric pressure). Furthermore, this paper presents the oxidation of some sulfides including DBT under mild conditions.

**Fig. 1:**
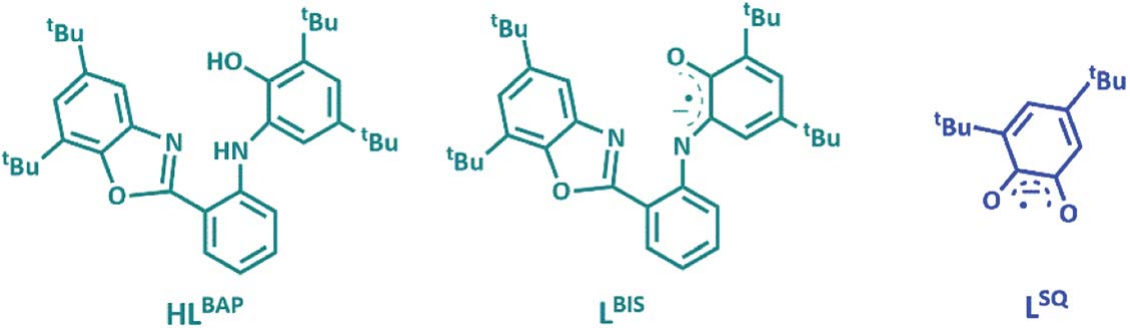
The ligand HL^BAP^, its one electron oxidized radical forms of L^BIS^ and its semiquinone hydrolyzed form L^SQ^.

The ligand HL^BAP^, its one electron oxidized radical form of L^BIS^ and its semiquinone hydrolyzed form L^SQ^, 3,5-di-*tert*-butyl benzosemiquinone are given in Fig. 1.

## Results and discussion

### Synthesis of MoOL^BIS^L^SQ^ complex

**Scheme 2:**
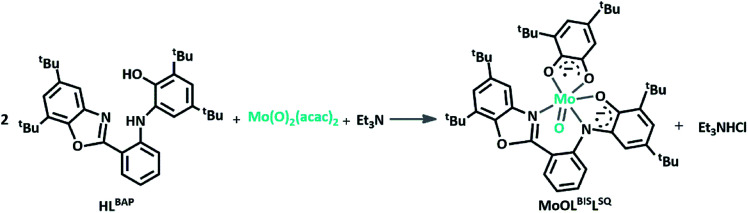
Synthesis MoOL^BIS^L^SQ^ complex from HL^BAP^, Mo(O)_2_(acac)_2_ and Et_3_N, (the L^SQ^ ligand in the resulted complex result from the hydrolysis of HL^BAP^).

The redox-active aminophenolate ligand H_2_L^BAP^ was synthesized from 1 : 2 molar ratio of 2-aminobenzyl amine and 3,5-DTBQ.^14^ The synthesis of MoOL^BIS^L^SQ^ complex was performed in 40% yield by refluxing CH_2_Cl_2_ solution of a ligand H_2_L^BAP^ and Et_3_N with MoO_2_(acac)_2_ (Scheme 2). The complex was purified by crystallization from CH_3_OH/CH_2_Cl_2_ 1 : 1 mixture and single crystals suitable for X-ray analysis were obtained after several recrystallizations.

Elemental analysis data of the complex are consistent with theoretical ones and confirm the 1 : 1 : 1 : 1 molar ratio of Mo : O : L^BIS^ : L^SQ^. The free ligand H_2_L^BAP^ shows characteristic IR bands at 3415 (*ν*_O–H_), 3258 (*ν*_N–H_), 1047 (C–N stretching), 1595 (C=C stretching), and 1542 cm^−1^ (C=N stretching), while *tert*-butyl group bands appear at 2962 cm^−1^ (Fig. S1†). In the IR spectrum of the complex, a sharp band is observed at 1026 cm^−1^, which is characteristic of the *ν*_Mo=O_ stretch,^15^ and the sharp and strong *ν*_O–H_ and *ν*_N–H_ absorptions of the ligand disappear, which confirms their coordination to the Mo(iv) center. All the vibrations of the ligand observed in the IR spectrum of the complex confirm the presence of the ligand in the structure (Fig. S2†).

### X-ray analysis

Crystallographic data of the complex are shown in Table 1. The Selected bond distances and angles are given in Table 2 and complete bond distances and angles and torsion angles [°] are presented in ESI Tables S1 and S2,† respectively.

**Table 1:** Crystallographic data for MoOL^BIS^L^SQ^

Empirical formula	C_49_H_64_MoN_2_O_5_
Formula weight	856.96
Crystal system	Triclinic
Space group	*P*−1
Unit cell dimensions	*a* = 10.6437(4), *b* = 14.3165(10), *c* = 16.2402(11)
	*α* = 96.269(6), *β* = 104.683(5), *γ* = 96.637(5)
Volume	2353.0(3) Å^3^
*Z*	2
Temperature	293(2) K
Density (calculated)	1.210 Mg m^−3^
Crystal size	0.531 × 0.385 × 0.167 mm^3^
Absorption coefficient	0.322 mm^−1^
Reflections collected	17 020
Independent reflections	10 489 [*R*(int) = 0.1080]
Goodness-of-fit on *F*^2^	1.039
Final *R* indices [*I* > 2sigma(*I*)]	*R*1 = 0.0887, w*R*2 = 0.2033
*R* indices (all data)	*R*1 = 0.1396, w*R*2 = 0.2544

**Table 2:** Selected bond lengths [Å] and angles [°] for MoOL^BIS^L^SQ^

Mo1–O4	1.993(4)	O5–Mo1–O3	159.75(15)
Mo1–O5	1.686(4)	N1–Mo1–N2	83.35(16)
Mo1–O1	1.955(4)	O3–Mo1–N2	79.43(16)
Mo1–N1	2.037(4)	O5–Mo1–O4	92.12(18)
Mo1–O3	2.091(4)	O4–Mo1–O1	86.52(15)
Mo1–N2	2.148(4)	O4–Mo1–N2	106.36(19)
C2–C3	1.383(9)	O1–Mo1–N2	158.35(16)
C4–C5	1.385(9)	N1–Mo1–O3	86.68(17)
O1–C1	1.340(7)	O1–Mo1–N1	78.86(16)
C6–N1	1.399(7)	O4–Mo1–O3	75.54(15)

**Fig. 2:**
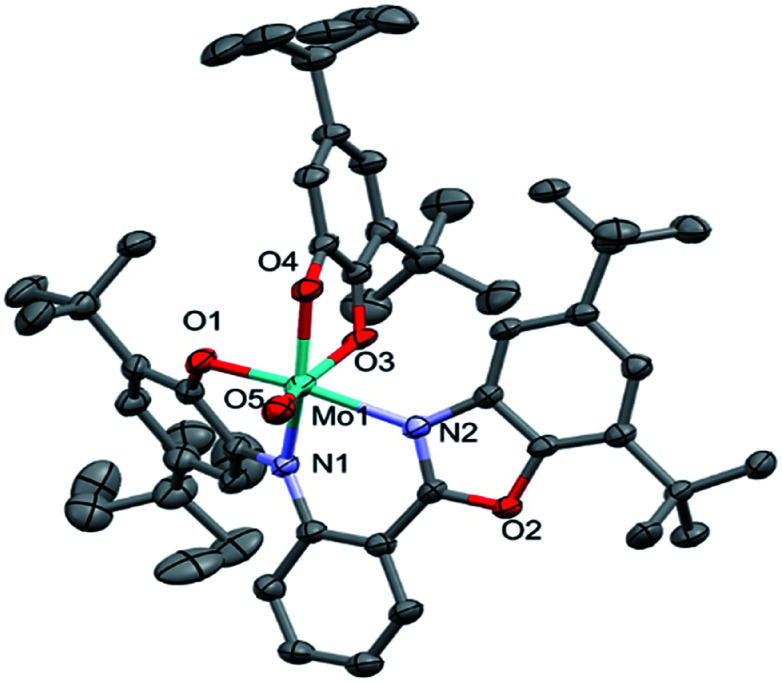
Molecular structure of MoOL^BIS^L^SQ^, hydrogen atoms have been omitted for clarity. The ADPs are plotted at 30% probability level.

The asymmetric unit of the structure contains the MoOL^BIS^L^SQ^ molecule (Fig. 2). The Mo(iv) oxidation form is assigned based on the presence of O^2−^ oxo, L^BIS 1−^ and L^SQ 1−^ (Fig. 1). The central Mo(iv) has a distorted octahedral coordination MoN_2_O_4_ sphere formed by tri-dentate L^BIS^ ligand (the oxidized form of L^AP^), the bi-dentate L^SQ^ and the oxo ligand. Similar coordination environment was also observed in complex VOL^BIS^(SQ).^16^

In the Mo coordination sphere, the shortest bond is the Mo1–O5 distance of 1.686(4) Å for the oxo ligand, while those of Mo1–O1, Mo1–O4 and Mo1–O3 are significantly longer, with the respective distances of 1.955(4), 1.993(4) and 2.091(4) Å. The Mo–N bonds formed by bridging N1 and the benzoxazole N2 are 2.037(4) and 2.148(4) Å, respectively. The L^SQ^ is coordinated to Mo in the hydrophobic cleft defined by di-*t*Bu-phenolic and di-*t*Bu-benzoxazole moieties of L^BIS^. Such a complex architecture significantly affects the geometry of the Mo coordination sphere, resulting in a distorted octahedral geometry.

In the L^BIS^ ligand, the C2–C3 and C4–C5 bond lengths are close to 1.38 Å, and are shorter than other endocyclic C–C bonds in the phenolic ring, which range from 1.40 to 1.42 Å (Table 2), suggesting at least partially localized double bonds. Both bonds involving imine N1 are similar in length and differ by approximately 1*σ*. Also, the O1–C1 and C6–N1 bonds are significantly shorter than single bonds.

Concerning the L^SQ^ ligand, both O3–C36 and O4–C41 are double bonds with the respective distances of 1.320(6) and 1.326(7) Å, while the C37–C38 and C39–C40 bonds are slightly shorter than the other endocyclic C–C bonds of the ligand (Table 2). All that suggests that L^BIS^ ligand is found in the semi-quinone form with some coupling between the phenolic and imine moieties.

### Magnetic susceptibility measurements

**Fig. 3:**
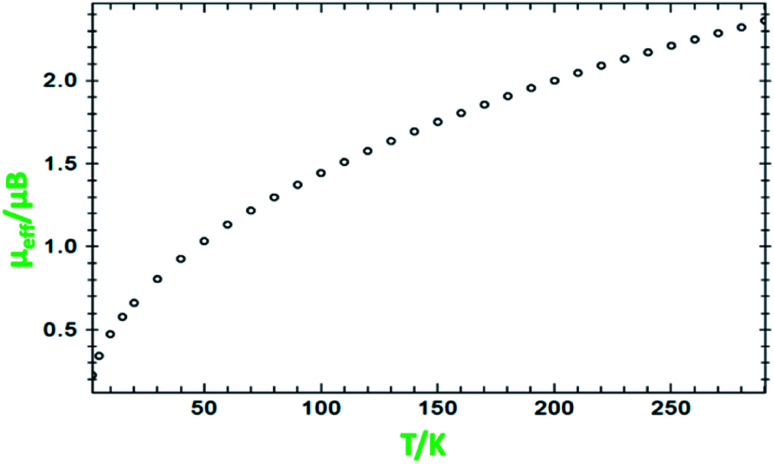
Variation of effective magnetic moment (*μ*_eff_) with variation in temperature for MoOL^BIS^L^SQ^.

**Scheme 3:**
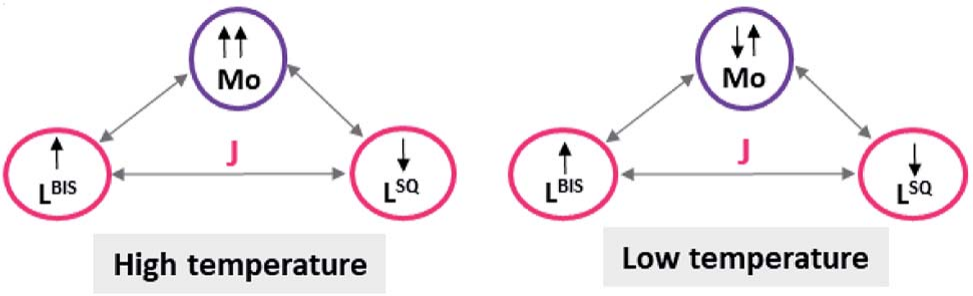
The schematic representation of spin distribution in MoOL^BIS^L^SQ^ at high and low temperature.

Variable-temperature magnetic susceptibility measurement for the crystalline samples of MoOL^BIS^L^SQ^ was performed with an applied magnetic field of 10 000 Oe in the temperature range 2–290 K (Fig. 3). The effective magnetic moment (*μ*_eff_) for MoOL^BIS^L^SQ^ at 290 K is 2.36 μ_B_, which is slightly lower than the spin-only value expected for two *S* = 1/2 radical spins. This value drops to almost zero (0.2 μ_B_) along with the decreasing temperature to 2 K indicating the ground singlet spin state (*S*_total_ = 0) and a dominant antiferromagnetic coupling in L^BIS^–Mo(iv)–L^SQ^ where spin alignment seems to be [(↓)–(↓↑)–(↑)] (Scheme 3). However, the temperature dependence of *μ*_eff_ is rather linear. Magnetic analysis by applying the spin system of two *S* = 1/2 was unsuccessful. These data are consistent with the X-ray structural data described above. Similar behavior was observed for the metal complex with a non-innocent ligand.^16^ Such behavior may be responsible for an unclear oxidation state of the Mo ion with redox-active ligands.

### Electrochemistry

**Fig. 4:**
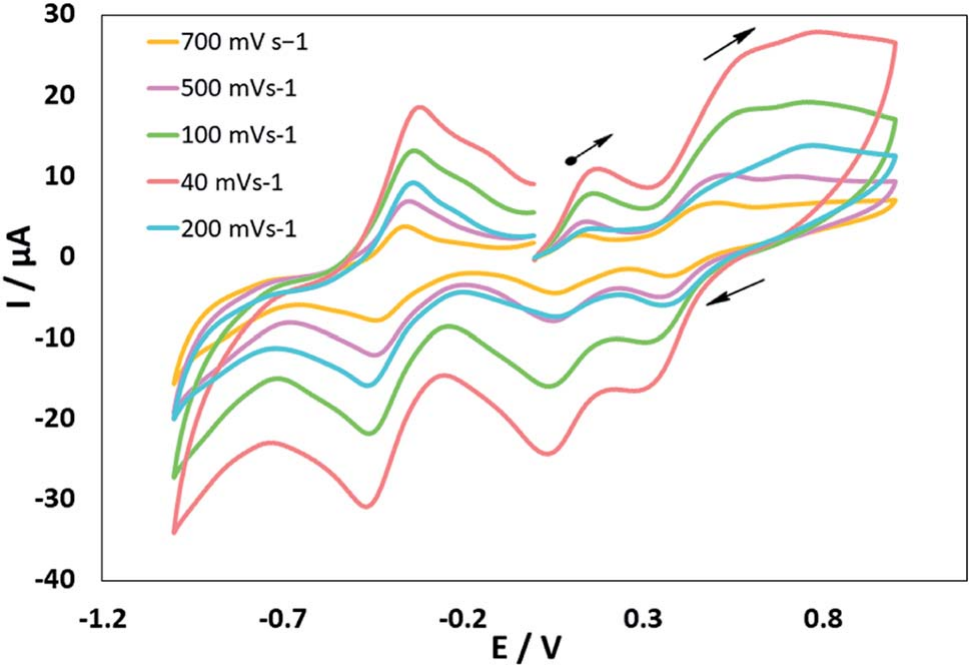
Cyclic voltammograms of MoOL^BIS^L^SQ^. Conds: 1 mM complex, 0.1 M NBu_4_ClO_4_, CH_2_Cl_2_, 298 K.

**Scheme 4:**
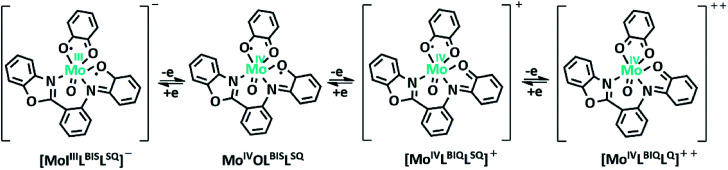
Schematic representation of MoOL^BIS^L^SQ^ complex oxidation state variation (*t*Bu groups are omitted for clearly).

The electrochemical properties of the MoOL^BIS^L^SQ^ complex were recorded in CH_2_Cl_2_ by cyclic voltammetry (CV) at a low temperature (233 K). Before starting the measurement, GC electrodes were polished with 0.1 mm alumina powder and washed with distilled water. CV reveals that there are three quasi-reversible redox peaks for the complex (Fig. 4). The complex underwent two quasi-reversible ligand centered one-electron redox processes, iminosemibenzoquinone L^BIS^/iminobenzoquinone L^BIQ^ and *o*-semibenzoquinone L^SQ^/*o*-benzoquinone L^Q^ redox couples at positive potentials, and Mo^IV^/Mo^III^ at negative potential as seen in eqn (1)–(3) (Scheme 4).^17^

1 Mo^IV^L^BIS^L^SQ^ ↔ Mo^IV^L^BIQ^L^SQ^

2 Mo^IV^L^BIQ^L^SQ^ ↔ Mo^IV^L^BIQ^L^Q^

3 Mo^IV^L^BIQ^L^Q^ ↔ Mo^III^L^BIQ^L^Q^

### Electronic spectroscopy

**Fig. 5:**
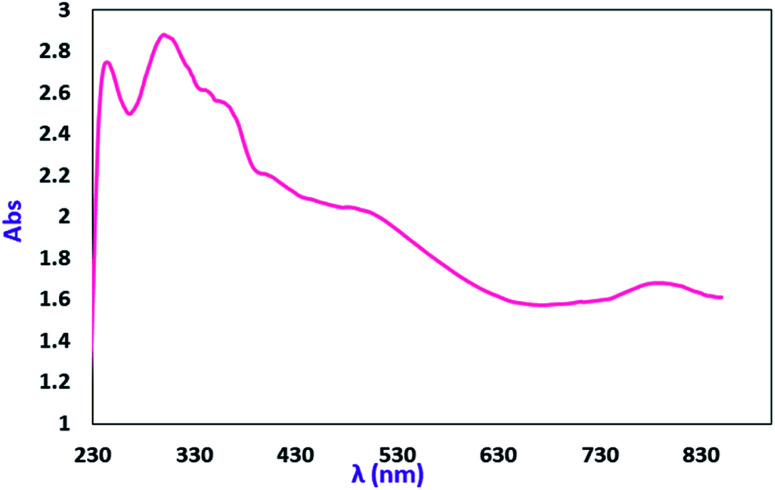
Electronic spectra of 2 mM CH_2_Cl_2_ solutions of MoOL^BIS^L^SQ^.

The electronic absorption (UV-vis/NIR) spectrum of the MoOL^BIS^L^SQ^ complex in dichloromethane is shown in Fig. 5. The spectra were recorded at room temperature (25 °C) in the range of 230–850 nm. The intense absorption bands in 246 and 296 nm in the higher energy and near-UV regions (below 320 nm) are resulted by π → π* transitions that involve iminosemiquinone, L^BIS^, and L^SQ^, units. The broad electronic absorption band in the region around 344–380 and 483–542 nm are consistent with iminosemiquinone ligands (L^BIS^ and L^SQ^)-to-Mo, (π)-to-Mo-(dπ*), the ligand to metal charge-transfer (LMCT). The absorption band that appeared at lower energy (788 nm) is due to d–d charge transfer.^17*a*^ The investigation of the theoretical analysis of the spectrum has been given below.

### DFT studies

**Fig. 6:**
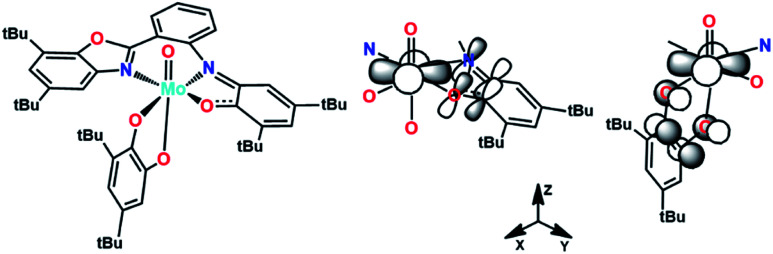
π-Donation to d_*xy*_ Mo orbital from the π_3_* orbital of (L^BIS^)^1−^ and (L^SQ^)^1−^ ligands.

To gain deeper understanding of complex [MoOL^BIS^L^SQ^], this species, the H_2_L^BAP^ compound and (L^BIS^)*^n^*^−^ and (L^SQ^)*^n^*^−^ ligands (*n* = 2, 1, 0), were theoretically analyzed by using the Density Functional Theory (DFT) approach at the B3LYP-LANL2DZ/6-311++G** level. The resulting optimized structure and HOMO of the precursor ligand H_2_L^BAP^ are shown in Fig. S3.† The theoretical description fits well with the X-ray experimental data previously reported for this compound.^14^ The HOMO of this species is centered on the π phenyl system and the distinctive π_3_* character is observed in the *o*-aminophenol moiety. This MO is responsible for the non-innocence behavior when it acts as ligand due to the π-donation from this orbital to the metal. Usually, this specific part is typically found as the main contribution to the SOMO of *o*-aminophenolate (−1) and benzosemiquinone (−1) type ligands. In fact, the characteristic antibonding combinations of C–N and C–O bonds and the C–C bonding combination were clearly observed in the HOMO of optimized (L^BIS^)^2−^ and (L^SQ^)^2−^ ligands or SOMO of (L^BIS^)^−^ and (L^SQ^)^−^ ligands (Fig. S4 and S5,† respectively). For a six-coordinated oxo-molybdenum(iv) complex, the LUMO description would be an empty d_*xy*_ orbital. Thus, the π-donation to this orbital from the (L^BIS^)^1−^ and (L^SQ^)^1−^ ligands is foreseeable as it is schematically shown in Fig. 6. This fits well with the observed bending of the metallacycle Mo–O1–C1–C6–N1 along the O1–N1 vector in the X-ray structure. This takes place for maximizing the overlap between the d_*xy*_ and π_3_* orbitals (white lobes in Fig. 6, center), as it has been previously noted in related ligands.^18^ Coordinates of the optimized compounds are listed in Table S3.†

**Fig. 7:**
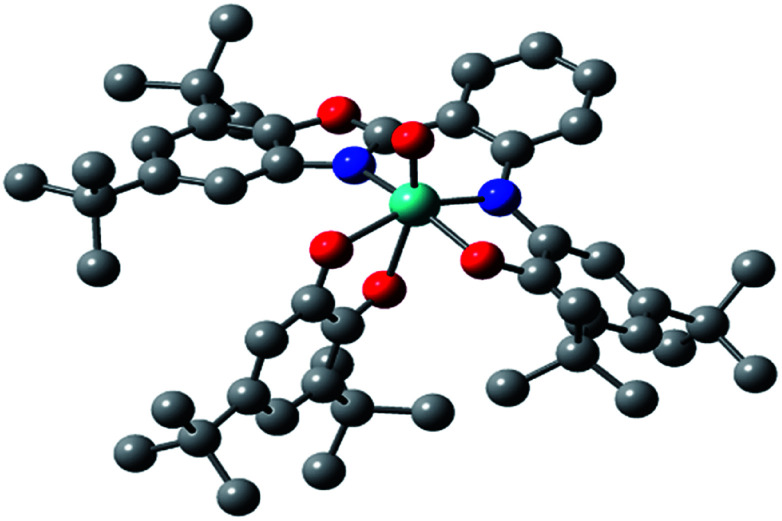
Optimized structure of complex MoOL^BIS^L^SQ^ (hydrogen atoms omitted for clarity).

**Fig. 8:**
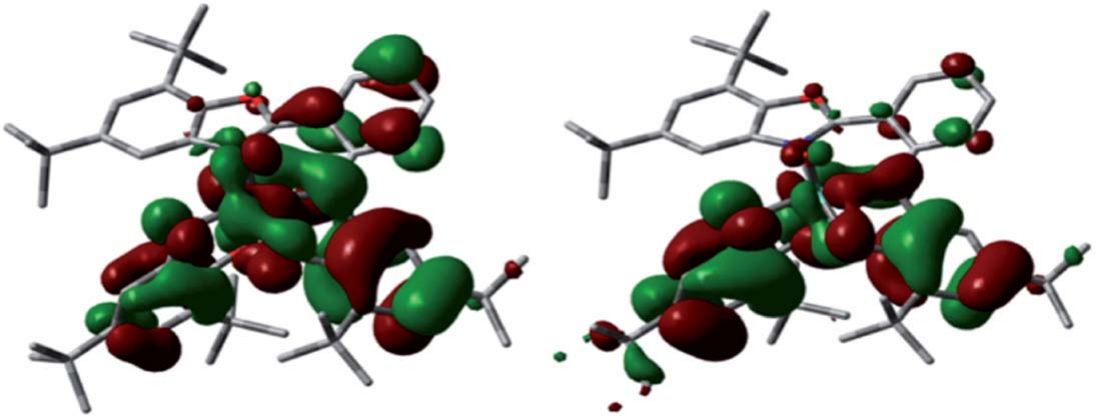
SOMO and SOMO−1 of complex MoOL^BIS^L^SQ^ (hydrogen atoms omitted for clarity).

Prior to the DFT study of MoOL^BIS^L^SQ^, free ligands (L^BIS^)*^n^*^−^ and (L^SQ^)*^n^*^−^ (*n* = 2, 1, 0) were analyzed. HOMO, SOMO and LUMO of these ligands are similar and their respective MO energies decreases from the dianionic to neutral species. Consequently, the dianionic ligand is the most suited for the π-donation to molybdenum d_*xy*_ orbital. Concerning the structural parameters, as expected, an enlargement of C=O and C=N– bond distances was observed going from neutral to dianionic ligand with a concomitant modification of phenyl C–C bonds (Fig. S4 and S5†). Based on these data and that of X-ray within the L^BIS^ and L^SQ^ ligands, the radical-anion form of these ligands was considered.^19^ Therefore, geometry optimization has been carried out assuming the presence of two unpaired electrons (*S* = 1) on ligands. The optimized MoOL^BIS^L^SQ^ complex describes the experimental structural parameters reasonably well (Fig. 7 and Table S4†). Fig. 8 displays the SOMO and SOMO−1 of this complex, in which SOMO−1 is mainly composed of L^BIS^ and L^SQ^ ligands (46 and 52%, respectively). SOMO involves the L^BIS^ ligand (55%) with 28% of L^SQ−^ and a small contribution of molybdenum (16%). Accordingly, both SOMOs are mostly centered on the (L^BIS^)^−^ and (L^SQ^)^−^ ligands confirming the proposed formulation. Additionally, the molecule was also optimized for *S* = 0 state, corresponding with the antiferromagnetic coupling observed at low temperature, and no significant structural changes were observed (see ESI†).

### TD-DFT calculation

To gain detailed insight into the absorption spectra and charge transitions, TD-DFT calculations were performed at the B3LYP level. The calculated excitation wavelength, oscillator strengths, and their assignment are given in Table S5.† The energies and compositions of molecular orbitals with a major contribution to charge transitions, are listed in Table S6.† Contour plots of these orbitals are shown in Table S7.† The six highest occupied molecular orbitals (HOMO, HOMO−2, HOMO−3, HOMO−4, HOMO−6, HOMO−8) of the Mo complex have large contributions (>90%) from L^BIS^ and L^SQ^ ligand π-bonding orbitals, while HOMO−1 consist of 25% Mo d-orbitals. As shown in Table S6,† the contribution of L^BIS^ and L^SQ^ ligands to the four lowest unoccupied orbitals (LUMO, LUMO+1, LUMO+2, LUMO+3) decrease and show great mixing of oxygen non-bonding, L^BIS^ and L^SQ^ ligands π* and Mo d-orbitals, while LUMO+1 mainly consist of L^BIS^ π* orbitals. The complex shows sharp bands at 246 and 296 nm in dichloromethane. These are mainly L^BIS^ ligand π-bonding to Mo d-orbitals/oxygen non-bonding orbitals charge transfer (ligand to metal or ligand to ligand (LLCT)). A shoulder appearing in the region around 344–380 nm can be assigned to the L^BIS^ to Mo/oxygen charge-transfer transition, while the low energy broad band located at 450–542 nm can be assigned predominately to the L^BIS^ ligand π-bonding orbitals to metal transition (Tables S5 and S6 of ESI†).

#### The catalytic activity of MoOL^BIS^L^SQ^ complex in oxidation reactions

##### Oxidation of cyclohexene to adipic acid

The catalytic behaviour of the MoOL^BIS^L^SQ^ complex in the oxidative cleavage of cyclohexene to adipic acid was investigated. A simple method was developed using 25% H_2_O_2_ as a green oxidant. After some screening experiments (Table 3), the highest yield of adipic acid was achieved after 10 h at 75 °C in the presence of MoOL^BIS^L^SQ^ (5 mol%) and H_2_O_2_ (5 eq.) (Table 3, entry 10). Under these conditions, the resulting white crystalline solid, was isolated by filtration and isolated, was identified as pure adipic acid (IR and mp at 151–152 °C). High selectivity was obtained according to the ^1^H NMR spectrum of the crude product which did not show any detectable by-products.

**Table 3:** The optimization condition for the oxidation of cyclohexene to adipic acid^a^

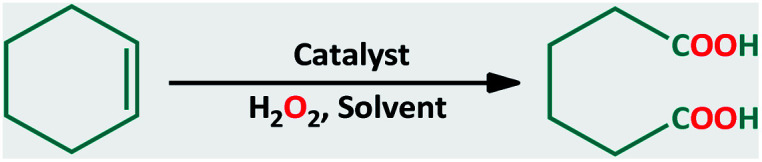						
	Catalyst (mol%)	Solvent	H_2_O_2_ (eq.)	Time (h)	Temp. (°C)	Yield^b^ (%)
1	3	Free	3	6	60	—
2	3	H_2_O	3	5	60	—
3	3	H_2_O/EtOH	3	6	60	10
4	3	EtOH	4	8	60	25
5	3	CH_2_Cl_2_	4	8	60	35
6	3	CH_3_CN	4	8	60	44
**7**	4	CH_3_CN	5	8	60	56
8	4	CH_3_CN	5	8	70	60
9	5	CH_3_CN	5	8	70	70
**10**	**5**	**CH**_**3**_ **CN**	**5**	**10**	**75**	**84**

a Reaction condition: cyclohexene (1 mmol), H_2_O_2_ (25%), catalyst (MoOL^BIS^L^SQ^), solvent (2 mL).

b Isolated yield.

**Scheme 5:**
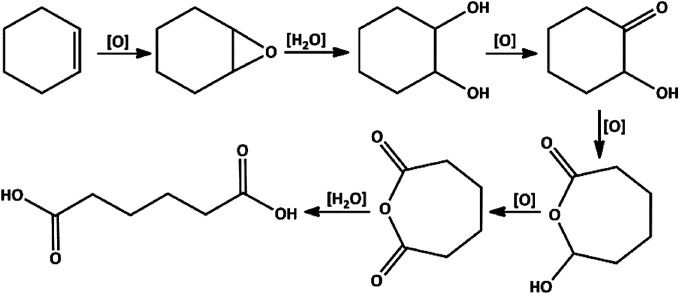
Proposed reaction pathway for cleavage oxidation of cyclohexene to adipic acid.

A suggested mechanism for the oxidation of cyclohexene to adipic acid is presented in Scheme 5.^20,21^ This mechanism consists of: (a) transformation of cyclohexene to 1,2-epoxycyclohexane, (b) 1,2-cyclohexanediol formation, (c) oxidation of the resulted diol to 2-hydroxycyclohexanone and then 7-hydroxyoxepan-2-one, and (d) adipic anhydride formation is the final intermediate which can simply go through the ring-opening in the acidic environment to produce adipic acid.

**Scheme 6:**
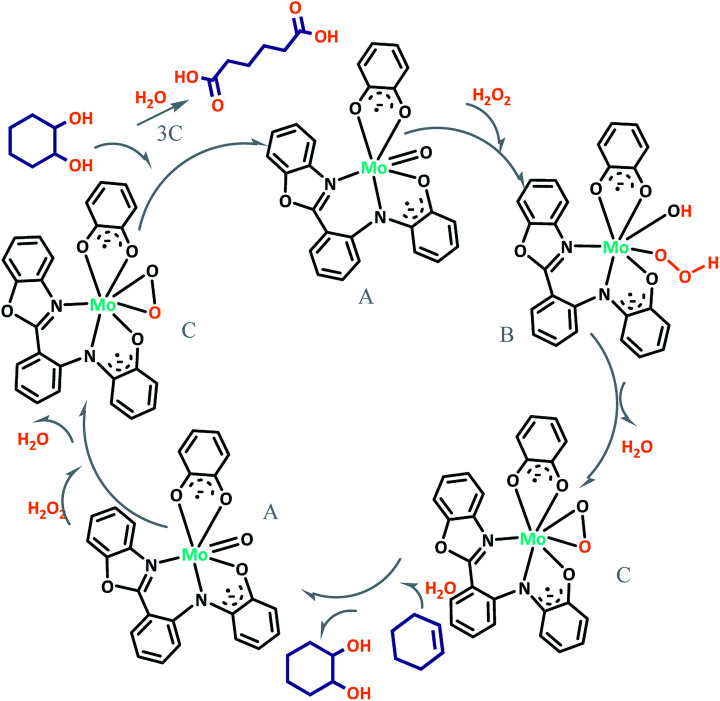
Proposed pathway for the formation of adipic acid.

The proposed mechanism of MoOL^BIS^L^SQ^ for the oxidation of cyclohexene to adipic acid is shown in Scheme 6. The reaction can be catalyzed by *in situ* development of peroxomolybdenum species from MoOL^BIS^L^SQ^ in acidic media. This active peroxo complex could undergo oxygen transfer to the substrate and produce epoxycyclohexane that in turn transforms to adipic acid *via* the steps which are shown in Scheme 5. After these steps, the recovered catalyst was subjected for another catalytic cycle.^20*c*^

Table 4 represents the comparison between the current work and some other previously reported literature procedures for the oxidative cleavage of cyclohexene to adipic acid by using different catalysts and reaction conditions.^20*b*,22–34^ Comparison shows that in other systems the reaction was performed using more expensive catalysts, longer time or stronger oxidant.

**Table 4:** Comparison of the results obtained from various methods for oxidation of cyclohexene oxidation to adipic acid

Catalyst	Oxidant	Time (h)	Temp. (°C)	AA (%)	Ref.
MoOL^BIS^L^SQ^	**H**_**2**_ **O**_**2**_	**10**	**60**	**84**	**This work**
Ag_2_WO_4_–IL 1	H_2_O_2_	18	75	85	22
[C_16_H_33_N(CH_3_)_3_]_2_W_2_O_3_(O_2_)_4_	H_2_O_2_	20	90	78	23
SSA@[BMIm]WO_4_^2−^	H_2_O_2_	18	75	87	24
SBA@Ti–Al	*t*-BuOOH	48	80	80	25
MIL-101	H_2_O_2_	8	70	90	26
InCl_3_	*t*-BuOOH	9	90	92	27
RuCl_3_	NaIO_4_	0.5	RT	90	28
[LSO_3_H]WO_4_^2−^	H_2_O_2_	12	87	85	20*b*
Na_2_WO_4_·2H_2_O [CH_3_(*n*-C_8_H_17_)_3_N]HSO_4_	H_2_O_2_	8	75–90	90	29
Na_2_WO_4_ + ILs	H_2_O_2_	10	Reflux	100	30
H_2_WO_4_ + IL	H_2_O_2_	12	73–87	85–96	31
H_4_SiW_12_O_40_	H_2_O_2_	US (25 kHz)	4	92	32
Ti-MMM-2Ce-SBA-15	H_2_O_2_	72	82	10–30	33
Ti-AlSBA-15	H_2_O_2_	24	70	80	34

##### Sulfide oxidation

**Scheme 7:**
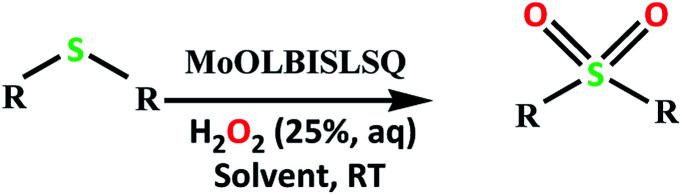
Selective oxidation of sulfides to sulfoxides or sulfones catalyzed by MoOL^BIS^L^SQ^.

The catalytic activity of the complex MoOL^BIS^L^SQ^ in the oxidation of some sulfides at room temperature under the reaction conditions stated in the Experimental section was also studied (Scheme 7).

First, we optimized conditions (catalyst amount, oxidant, and solvent) for the transformation of sulfides to sulfoxide and sulfone, using the oxidation of methyl phenyl sulfide as a model reaction (Table 5).

**Table 5:** The optimization condition for the oxidation of methyl phenyl sulfide

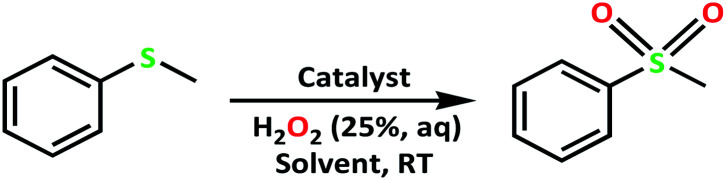						
Entry	Catalyst (mol%)	Solvent	H_2_O_2_ (eq.)	Time (h)	Conversion^a^ (%)	TON^b^
1	1	EtOH	2	5	50	50
2	1	EtOH	3	5	70	70
3	2	EtOH	3	4	94	47
4	2	EtOH	4	4	100	50
5	2	H_2_O	3	6	15	75
6	2	EtOH/H_2_O	3	6	30	15
7	2	Toluene	3	4	45	22.5
8	2	Free	3	4	50	25
9	2	Free	4	4	56	28
10	3	Free	4	5	76	25.3
**11**	**2**	**Acetone**	**3**	**4**	**100**	**50**

a Sulfide to sulfone conversions were determined by using GC.

b TON = (substrate/catalyst) × conversion.

The effect of several solvents, such as EtOH, H_2_O, *etc.*, was studied revealing that acetone and ethanol (Table 5, entry 4, and 11) appear to be the most favorable solvents. The free solvent condition was then investigated and a good result was also achieved (Table 5, entry 10).

We also checked the amount of H_2_O_2_ for the oxidation of methyl phenyl sulfide at room temperature and realize that the best catalytic performance was achieved by using 3 and 4 eq. of H_2_O_2_ in the case of acetone and ethanol respectively (Table 5, entries 4 and 11). Then we studied the effect of the amount of catalyst between 1 mol% and 3 mol% and the best results were reached when the amount of the catalyst was 2 mol%. As it is obvious in Table 5, the catalytic activity of the MoOL^BIS^L^SQ^ depends on the amount of catalyst and oxidant considerably.

After determining under optimized reaction conditions for sulfoxidation of methyl phenyl sulfide (substrate: 1 mmol, catalyst: 2 mol%, acetone 2 mL, H_2_O_2_: 3 eq., time: 4 h and room temperature), the catalytic activity of the MoOL^BIS^L^SQ^ was studied in the oxidation of different sulfides under these optimized parameters (Table 6).

**Table 6:** Oxidation of various sulfides to sulfone with 25% H_2_O_2_ catalyzed by MoOL^BIS^L^SQ^^a^

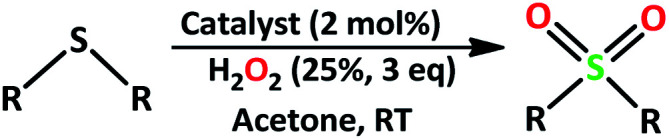					
Entry	Substrate	Time (h)	Conversion^b^ (%)	Selec. to sulfone^c^ (%)	TON^d^
1	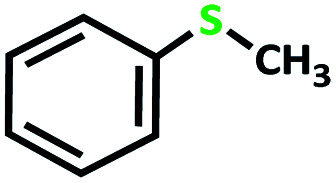	4	100	>99	50
2	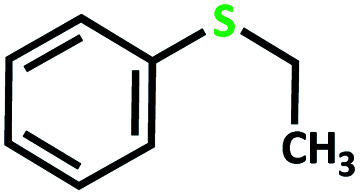	4	100	>99	50
3	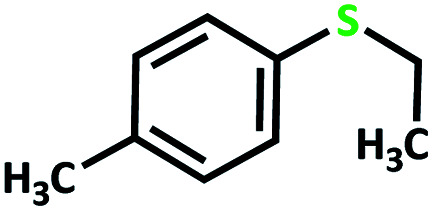	4	100	>99	50
4	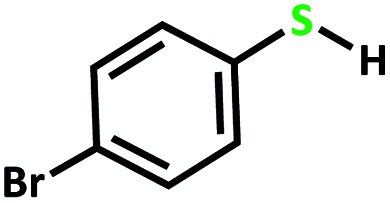	5	66	>99	33
5	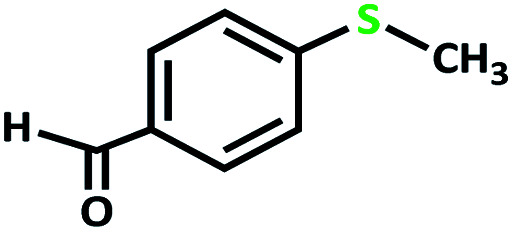	4	100	>99	50
6	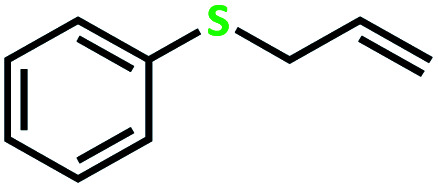	4	>99	>99	49
7	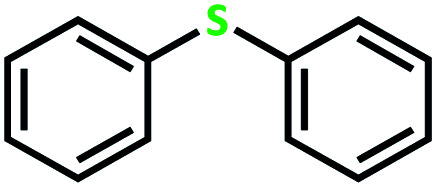	5	84	>99	42
8	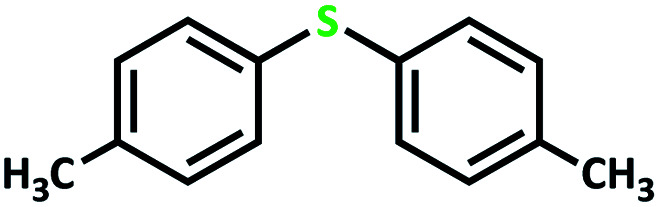	4	>99	>99	49.5
9	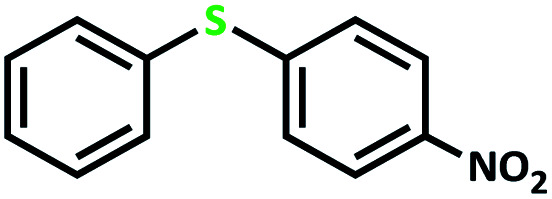	5	88	>99	44
10	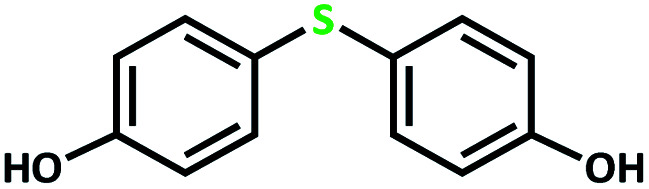	5	>99	>99	49.5
11	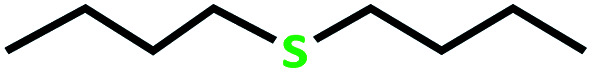	5	91	>99	45.5
12	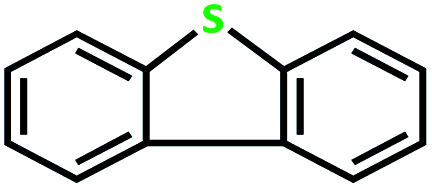	6	50^e^	>99	25

a Reaction conditions: catalyst : sulfide : H_2_O_2_ = 0.02 : 1 : 3 in 2.5 mL acetone.

b Sulfide to sulfone conversions were determined by using GC.

c Selectivity to sulfone = [*A*%/(*A* + *B*%)] × 100.

d TON = (moles of substrate/moles of catalyst) × conversion.

e Isolated yield.

The catalyst performance is good for various sulfides. It has to be stated that the oxidation of DBT (dibenzothiophene) did not happen in any measurable amount in the optimized conditions. New reactions for DBT with a stronger oxidant and TBHP (*tert*-butyl hydroperoxide or *t*-BuOOH) were studied. These results are summarized in Table 7. We can see the oxidation of DBT using MoOL^BIS^L^SQ^ with the efficient conversion of 90% and selectivity of >99 and TON of 30 (Table 7, entry 10).

**Table 7:** Oxidation of DBT with TBHP catalyzed by MoOL^BIS^L^SQ^

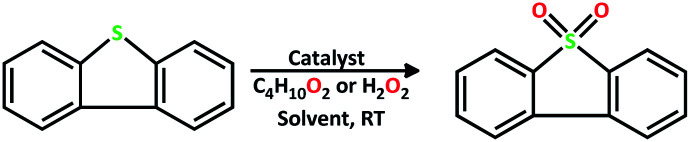							
Entry	Catalyst (mol%)	Solvent	Oxidant (eq.)	Time (h)	Conversion^a^ (%)	Selec. to sulfone^b^ (%)	TON^c^
1	2	Acetone	H_2_O_2_ (3)	5	10	>99	5
2	2	Acetone	H_2_O_2_ (4)	5	10	>99	5
3	3	Acetone	H_2_O_2_ (4)	5	15	>99	5
4	3	EtOH	H_2_O_2_ (4)	4	15	>99	5
5	2	Acetone	H_2_O_2_ (excess)	4	15	>99	7.5
6	2	Acetone	TBHP (2)	4	75	>99	37.5
7	2	Free	TBHP (2)	4	50	>99	25
8	2	EtOH	TBHP (2)	4	60	>99	30
9	2	Toluene	TBHP (2)	5	80	>99	40
**10**	**3**	**Toluene**	**TBHP (2)**	**5**	**90**	**>99**	**30**
8	3	Toluene	TBHP (3)	5	90	>99	30

a The conversions were determined by GC.

b Selectivity to sulfone = [*A*%/(*A* + *B*%)] × 100.

c TON = (substrate/catalyst) × conversion.

Finally, the role of the catalyst and also the effect of metal–ligand synergistic effects proved by some blank tests with H_2_O_2_, MoO_2_(acac)_2_/H_2_O_2_, and H_2_L^BIS^/H_2_O_2_ catalytic systems and the results are collected in Table S8.†

To improve the understanding of the reaction mechanism, sulfide was added to the stirring solution of MoOL^BIS^L^SQ^. No obvious and noticeable change was observed in the reaction mixture even after 2 h of stirring (Table S8,† entry 5). This result proved the essential presence of H_2_O_2_ to activate the complex.

**Scheme 8:**
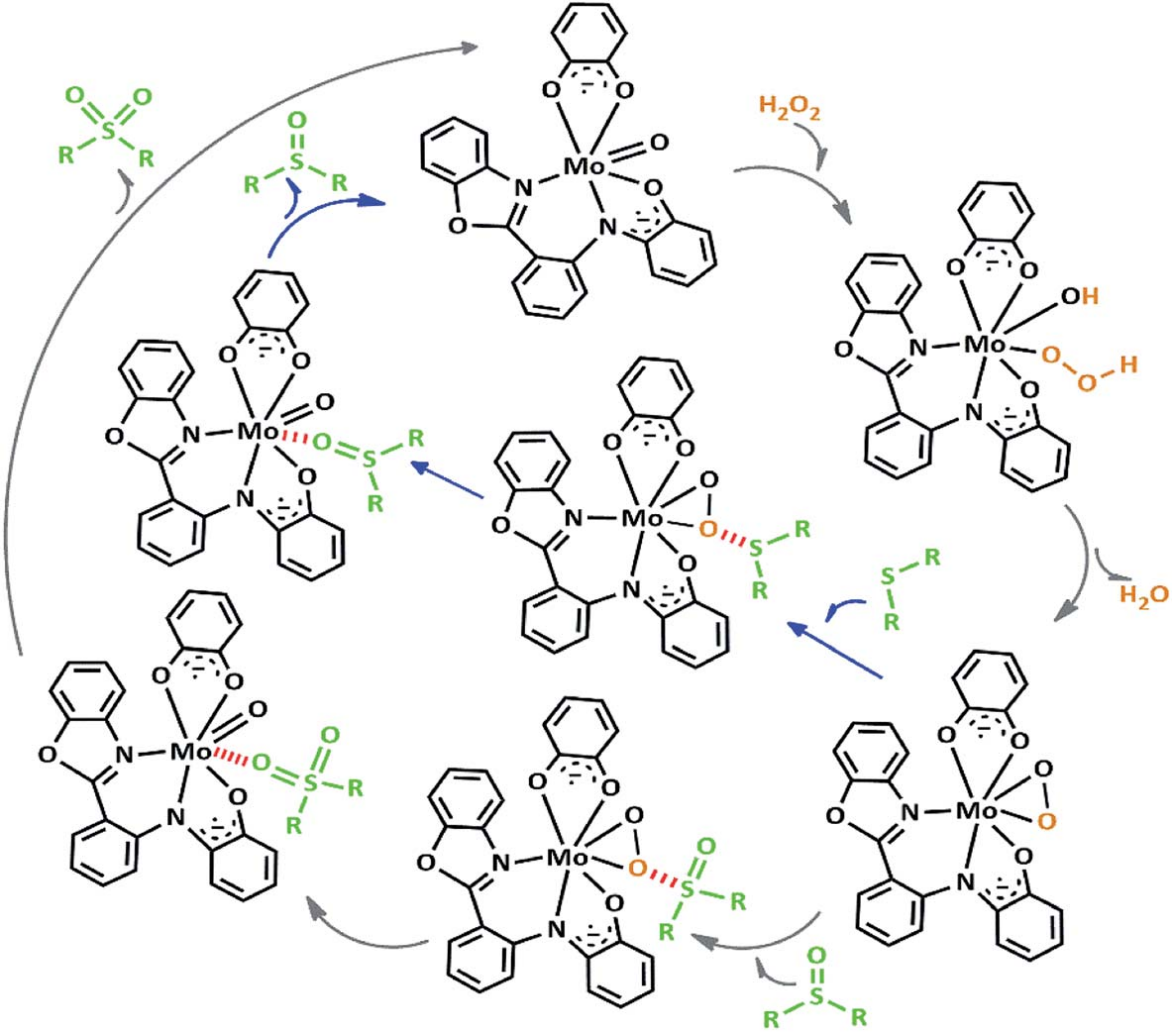
Proposed pathway for the formation of sulfoxide and sulfone.

We proposed a plausible mechanism for this oxidation reaction which is presented in Scheme 8. In the first step, the peroxo complex of the catalyst MoOL^BIS^L^SQ^ was achieved in the presence of H_2_O_2_, and these active spices are involved in the sulfoxidation reaction. The oxotransfer from the peroxo complex could be achieved in two ways. One is by direct oxygen transfer and the other is the coordination of the sulfide to the molybdenum. We proposed that due to the absence of free coordination position in the complex, the second idea seems to be more plausible. Thus, the oxygen transfer took place and the former complex recovered. Finally, sulfone could be obtained by another oxygen transfer to the resulted sulphoxide.^35^

Some literature data for the oxidation of sulfides with homogeneous and heterogeneous Mo-based catalysts are listed in Table 8. Comparison proves that the developed catalyst shows a good affinity for sulfide oxidation at mild condition comparing other reports.^35–47^

**Table 8:** Reaction conditions for the oxidation of sulfides with some Mo-based catalysts

	Catalyst (mol%)	Sulfide	Reaction condition: oxidant (mmol)/*T* (h)/temp. (°C)	AA (%)	Ref.
1	[MoO_2_(CH_3_OH)H_2_O(CH_3_OH)] (5)	Thioanisole	H_2_O_2_ (1)/13 h/0	83	2011 (ref. 35)
2	[MoO(O_2_)_2_(H_2_O)_n_]/HLiPr/[PPh_4_]Br (2.5)	Thioanisole	H_2_O_2_ (1)/1 h/0/reactor	95	2018 (ref. 36)
3	Mo(O_2_)L (5) L = 4,6-*O*-ethylidene-*N*-(2-hydroxybenzylidene)-β-d-glucopyranosylamine	Thioanisole	UHP (1)/15 h/RT	86	2016 (ref. 37)
4	MoO_2_(acac)_2_	Thioanisole	TBHP/—/50–70	98	1966 (ref. 38)
5	MoO_2_Cl_2_ (15)	Aryl sulfides	H_2_O_2_ (4)/8 h/RT	93	2006 (ref. 39)
6	MoO_2_Cl_2_ (4,4′-di-*tert*-butyl-2,2′-bipyridine)	S in diesel	H_2_O_2_/3 h/50	76	2016 (ref. 40)
7	[MoO_2_(L) (CH_3_OH)] (5)	Thioanisole	UHP/0.5 h/RT	92	2009 (ref. 41)
8	(NH_4_)6Mo_7_O_24_·4H_2_O (10)	Aliphatic and aryl sulfides	H_2_O_2_ (4)/45 min/RT	95	2009 (ref. 42)
9	[(*n*-C_4_H_9_)_4_N]_4_(α-Mo_8_O_26_)	Thioanisole	H_2_O_2_ (1)/10 min/RT	99	2009 (ref. 43)
10	Na_3_[CrMo_6_O_24_H_6_]·8H_2_O (2)	Thioanisole	H_2_O_2_ (2)/10 h/60	94	2010 (ref. 44)
11	(PyH)(H_3_PMo_11_VO_40_) (1)	Thioanisole	H_2_O_2_ (20)/2.5 h/40	95	2011 (ref. 45)
12	(CTA)_2_[MoO(O_2_)2(C_2_O_4_]·H_2_O (2.5)	Thioanisole	H_2_O_2_ (1)/30 min/95	95	2012 (ref. 46)
13	Mo@imine-Z	Diphenyl sulfide	H_2_O_2_ (2.5)/30 h/RT	95	2018 (ref. 47)
14	MoOL^BIS^L^SQ^ (2)	**Thioanisole**	**H**_**2**_ **O**_**2**_ **(4)/4 h/RT**	**100**	**This work**

## Experimental

### Materials

All the chemical compounds and solvents were purchased from commercial companies and used as received, except those for electrochemical measurements. 3,5-Di-*tert*-butylcyclohexa-3,5-diene-1,2-dione (3,5-DTBQ) was synthesized according to the literature procedure.^48^

### Synthesis of H_2_L^BAP^

Ligand (H_2_L^BAP^), 2,4-di-*tert*-butyl-6-(2(5,7-di-*tert*-butylbenzo[*d*]oxazol-2-yl) phenylamino) phenol, was synthesized with the procedure reported in the literature.^14^ Yield: 0.418 g (79%). Anal. calcd (found) for C_35_H_46_N_2_O_2_: C 79.71 (79.56), H 8.74 (8.51), N 5.30 (5.36). *ν*_max_ (KBr)/cm^−1^: 3415 (O–H), 3258 (N–H), 2959 (C–H), 1592 (C=C), 1542 (C=N), 1262 (C–O), 1047 (C–N) (Fig. S1†).

### Synthesis of MoOL^BIS^L^SQ^

To a solution of H_2_L^BAP^ (0.262 g; 0.5 mmol) in CH_2_Cl_2_ (5 mL), [Mo(O)_2_(acac)_2_] (0.164 g; 0.5 mmol) was added under an argon atmosphere and refluxed for 3 hours. Then the solution was stirred in air at room temperature for 1 hour. The resulting dark violet solution was then mixed with CH_3_OH and filtered. Dark violet micro crystals obtained after two days and single crystals suitable for X-ray analysis were obtained after several recrystallizations from CH_3_OH/CH_2_Cl_2_ 1 : 1 mixture. Yield: 0.350 g (40%). Anal. calcd (found) for C_49_H_64_MoN_2_O_5_: C 68.71 (67.91), H 7.47 (7.71), N 3.26 (3.46). *ν*_max_ (KBr)/cm^−1^: 2955 (C–H), 1650 (C=C), 1519 (C=N), 1365 (C–O), 1164 (C–N) (Fig. S2†).

### General procedure for investigating the catalytic activity of MoOL^BIS^L^SQ^ complex in the cyclohexene oxidation

In a typical procedure: MoOL^BIS^L^SQ^ (4 mol%, 0.034 g), cyclohexene (1 mmol) and 4 eq. of 25% H_2_O_2_ were mixed in a 25 mL round bottomed flask. Then the mixture was vigorously stirred at room temperature for 10 minutes and stirred at 70 °C for required time. The white precipitate was separated *via* filtration and then washed with a little volume of cold water to give adipic acid which was identified by ^1^H NMR (400 MHz, DMSO-*d*_6_): 1.50 (t, 4H), 2.25 (t, 4H), 12.10 (s, 2H) (Fig. S6†).^20^

### General procedure for determination the catalytic activity of MoOL^BIS^L^SQ^ complex in the sulfide oxidation

In a typical experimental procedure, a mixture of sulfide (1 mmol), H_2_O_2_ (3 eq., 25%), MoOL^BIS^L^SQ^ (2 mol%, 0.017 g) were firstly added to a 25 mL round bottom flask and stirring was continued for the required time (Table 3) at room temperature. TLC monitored the progress of the reaction (*n*-hexane : ethyl acetate, 5 : 1). After completion of the reaction, the conversion of sulfide and selectivity were determined by GC.

In the case of dibenzothiophene sulfone isolated yield has been reported. ^1^H NMR (400 MHz, chloroform-*d*) *δ* 7.90–7.79 (m, 4H), 7.67 (td, *J* = 7.60, 1.19 Hz, 2H), 7.56 (td, *J* = 7.58, 1.07 Hz, 2H). ^13^C NMR (101 MHz, CDCl_3_) *δ* 121.60, 122.20, 130.40, 131.62, 133.91, 137.71 (Fig. S7 and S8†).^16^

The conversion of the reaction was then calculated from the sulfide, sulfoxide and sulfone area signals by following formula:

% conversion = 100 × ([product])/([substrate] + [product]).

### Measurements

Dark violet crystals of MoOL^BIS^L^SQ^ were grown from the CH_3_OH : CH_2_Cl_2_ 1 : 1 solution. Diffraction data for this complex were collected with the Oxford Sapphire CCD diffractometer with graphite-monochromated MoKα radiation (*λ* = 0.71073 Å) at 292(2) K, by the *ω*–2*θ* method. The structure was solved using the Patterson method and refined by the full-matrix least-squares method on *F*^2^ with the SHELX2017 program package.^49^ Analytical absorption correction was applied by the RED171 package of programs^50^ Rigaku OD, 2015, and the minimum and maximum transmission of 0.957 and 0.905. Hydrogen atoms were positioned with electron density maps and constrained in the refinement.

Elemental analyses (C, H, and N) were done by Elementar Vario EL III. Fourier transform infrared spectroscopy on KBr pellets was performed with FT IR Bruker Vector 22 instrument. ^1^H NMR spectra were performed on a Bruker DRX instrument in CDCl_3_ at 400 MHz. The chemical shifts were referred to as TMS by residual signals from the solvent. Cyclic voltammetry (CV) was acquired on a PAR-263A potentiometer analyzer equipped with 0.1 M NBu_4_ClO_4_ solutions in CH_2_Cl_2_ as supporting electrolyte and with an Ag wire as the reference electrode, a glassy carbon as the working electrode, a Pt counter electrode, and also ferrocene internal standard. UV-vis absorbance spectra were recorded on a CARY 100 Bio spectrophotometer. The magnetic measurements were performed using a Quantum Design SQUID magnetometer MPMS-XL between 1.8 and 300 K. Measurements were done on a polycrystalline sample of 35 mg for MoOL^BIS^L^SQ^.

### Computational details

The electronic structure and geometry optimized of MoOL^BIS^L^SQ^ complex, H_2_L^BAP^ compound, and (L^BIS^)*^n^*^−^ and SQ*^n^*^−^ ligands (*n* = 2, 1, 0) were computed using density functional theory at the B3LYP level.^51^ The Mo atom was described with the LANL2DZ basis set^52^ while the 6-31G** basis set was initially used for C, O, N and H atoms. Optimized geometries of all the compounds were characterized as energy minima by the nonexistence of imaginary frequencies (NImag = 0) in the diagonalization of the analytically computed Hessian (vibrational frequencies calculations). The resulting geometries were further optimized at the LANL2DZ/6-311++G** level of theory for obtaining a better MO description. DFT calculations were achieved using the Gaussian 09 suite of programs.^53^ Coordinates of the optimized compounds are given in Table S3 (ESI†).

The solvent was included in the geometry optimization and TD-DFT computations utilizing the Polarizable Continuum Model (PCM)^54^ using dichloromethane (CH_2_Cl_2_) as a solvent with dielectric constant *ε* = 8.94.

## Conclusion

In this work, complex MoOL^BIS^L^SQ^ was synthesized and characterized. This complex is a distorted octahedral mononuclear molybdenum(iv) complex with the MoN_2_O_4_ sphere and with the coordinating ligands L^BIS^ and L^SQ^ present in the one-electron oxidized *o*-iminobenzosemiquinone [(ISQ)^1−^] forms. The complex acts as an efficient and environmentally friendly catalyst under mild conditions for oxidative cleavage of cyclohexene using H_2_O_2_ as an oxidant. One of the interesting features of this work lies with high selectivity toward adipic acid. A comparison of the adipic acid production with the catalyst reported here and other complexes shows the good activity of this complex. The catalyst was also efficient and selective for the oxidation of sulfides, especially DBT. The surrounding non-innocent ligands have a strong effect on the total electronics and catalytic reactivity of the complex.

## Conflicts of interest

There are no conflicts to declare.

## Supplementary Material

RA-010-D0RA06351G-s001

RA-010-D0RA06351G-s002
