# Supplementary material for: A biradical oxo-molybdenum complex containing semiquinone and o-aminophenol benzoxazole-based ligands
Source: RSC Adv. 2020 Nov 9;10(67):40853–66. doi: 10.1039/d0ra06351g (PMC9059147; doi:10.1039/d0ra06351g)
Supplement: RA-010-D0RA06351G-s001 [file RA-010-D0RA06351G-s001.pdf]

Electronic Supplementary Material (ESI) for RSC Advances.
This journal is © The Royal Society of Chemistry 2020

Supporting Information

Biradical Oxo-molybdenum Complex Containing a Semiquinone and *O*-aminophenol Benzoxazole-Based Ligands: Cyclohexene and Sulfide Catalytic Oxidation

Mina Nasibipour [a], Elham Safaei [a,*], Andrzej Wojtczak [b], Zvonko Jaglicˇic´ [c], Agustín Galindo [d], Marzieh Masoumpour [e]

[a] Department of Chemistry, College of Sciences, Shiraz University, 71454, Shiraz, Iran
[b] Nicolaus Copernicus University, Faculty of Chemistry, 87-100 Torun, Poland
[c] Institute of Mathematics, Physics and Mechanics & Faculty of Civil and Geodetic Engineering, University of Ljubljana, Jadranska 19, Ljubljana, Slovenia
[d] Departamento de Química Inorgánica, Facultad de Química, Universidad de Sevilla, Aptdo. 1203, 41071 Sevilla, Spain
[e] Department of Chemistry, Estahban Higher Education Center, Estahban 74519-44655, Iran

Corresponding Author
Email: e.safaei@shirazu.ac.ir

contents	page
**Figure S1** IR spectrum of H_2L^{BAP}	S3
**Figure S2** IR spectrum of $MoOL^{BIS}L^{SQ}$	S3
**Table S1** Bond distances (Å) and bond angles (?)	S4
Crystal information of complex $MoOL^{BIS}L^{SQ}$	S5
**Table S2** Torsion angles [°] for $MoOL^{BIS}L^{SQ}$	S6
**Figure S3** Optimized structure and HOMO (hydrogen atoms omitted for clarity) of compound H_2L^{BAP}	S8
**Figure S4** Selected bond lengths, MOs (hydrogen atoms omitted for clarity) and energies of optimized $(L^{BIS})^{n-}$ ligands (n = 2, 1, 0)	S8
**Figure S5** Selected bond lengths, MOs (hydrogen atoms omitted for clarity) and energies of optimized SQ^{n-} ligands (n = 2, 1, 0)	S8
**Table S3** Coordinates of the optimized compounds	S9
**Table S4** Comparison of experimental and theoretical structural parameters of complex $MoOL^{BIS}L^{SQ}$	S20
**Table S5** Experimental and selected list of calculated absorption properties of the $MoOL^{BIS}L^{SQ}$ complex	S21
**Table S6** Energies (eV) and composition (%) of some frontier molecular orbitals of $MoOL^{BIS}L^{SQ}$ complex	S21
**Table S7** Molecular orbitals corresponding to TD-DFT excitations for the $MoOL^{BIS}L^{SQ}$ complex	S22
**Table S8** The result of blank test for oxidation of phenyl sulfide with some catalytic systems	S23
**Figure S6** ^{1}H NMR spectrum of adipic acid	S24
**Figure S7** ^{1}H NMR spectrum of dibenzothiophene sulfone	S25
**Figure S8** ^{13}C NMR spectrum of dibenzothiophene sulfone	S26

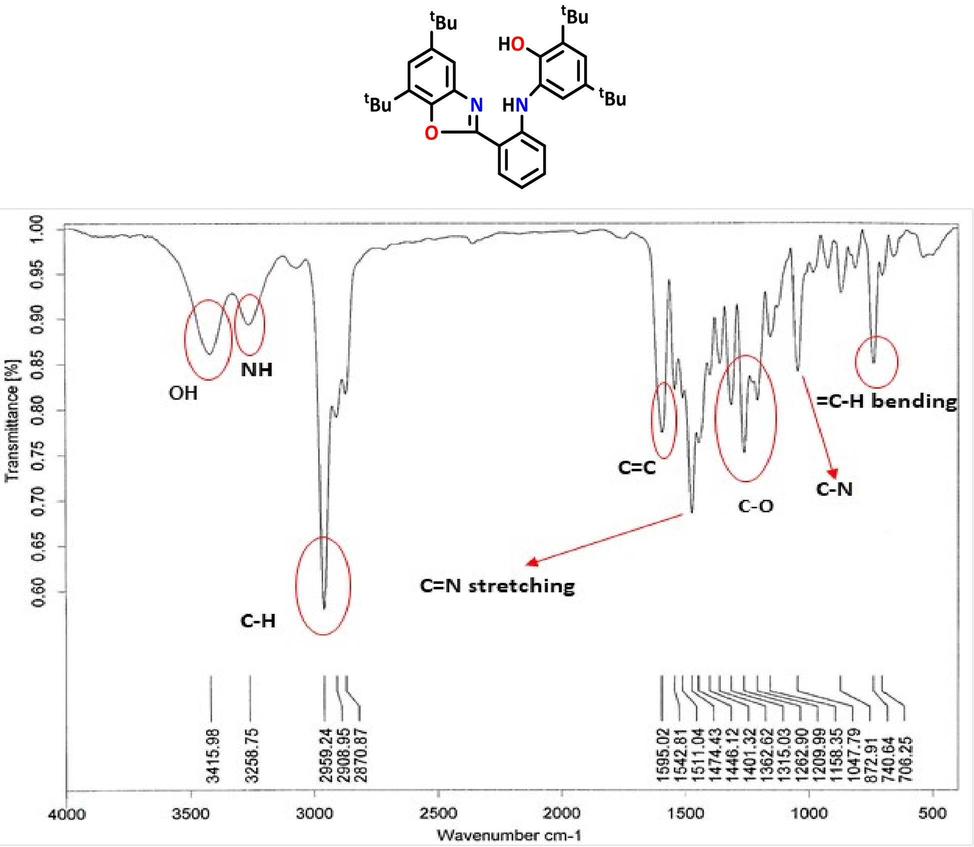

Figure S1 IR spectrum of H_2L^{BAP}

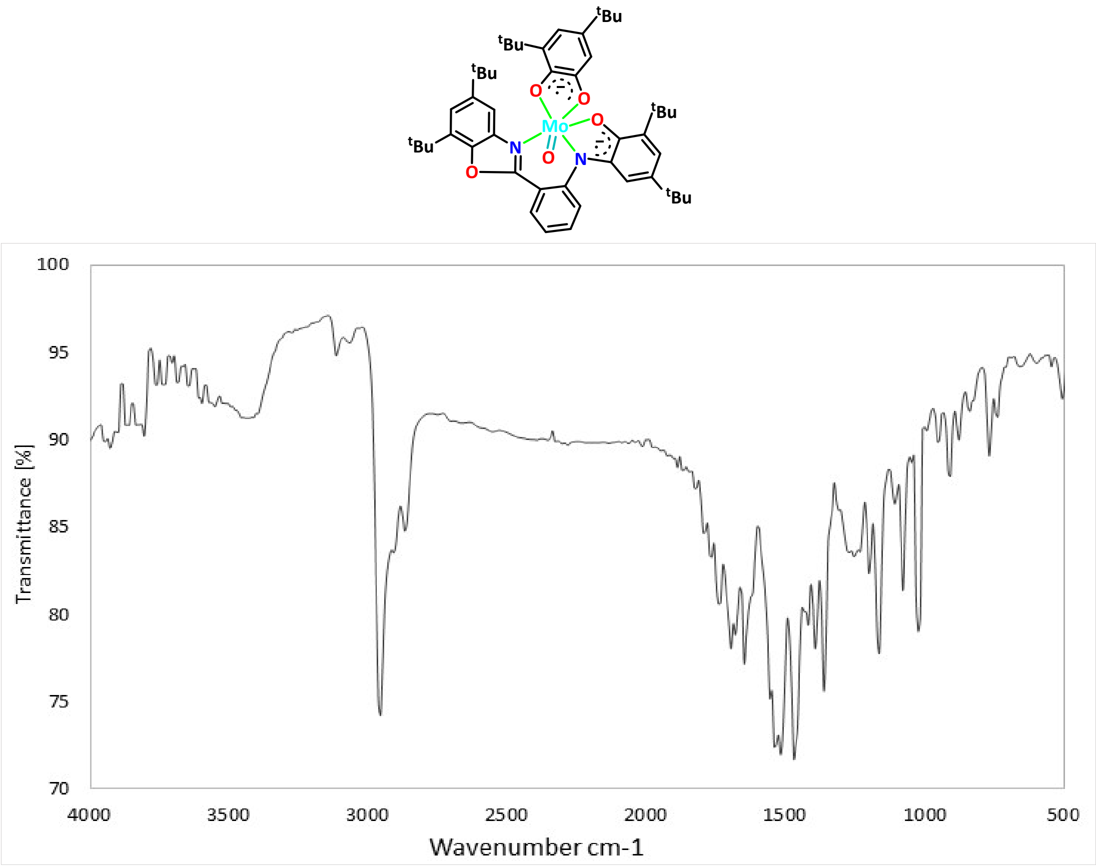

Figure S2 IR spectrum of $MoOL^{BIS}L^{SQ}$

Table S1 Selected bond lengths [Å] and angles [°] for MoOL^{BIS}L^{SQ}

Mo1-O4	1.684(4)	O4-Mo1-O1	108.18(18)
Mo1-O1	1.956(4)	O4-Mo1-N3	92.28(19)
Mo1-N3	1.986(4)	O1-Mo1-N3	86.50(16)
Mo1-N1	2.039(4)	O4-Mo1-N1	108.4(2)
Mo1-O3	2.090(4)	O1-Mo1-N1	78.77(16)
Mo1-N2	2.150(4)	N3-Mo1-N1	157.50(18)
O1-C1	1.340(7)	O4-Mo1-O3	159.76(18)
C1-C2	1.404(8)	O1-Mo1-O3	87.33(16)
C1-C6	1.418(8)	N3-Mo1-O3	75.35(15)
C2-C3	1.383(9)	N1-Mo1-O3	86.96(17)
C2-C20	1.549(8)	O4-Mo1-N2	89.04(18)
C3-C4	1.419(9)	O1-Mo1-N2	158.34(17)
C4-C5	1.386(9)	N3-Mo1-N2	106.35(15)
C4-C24	1.529(9)	N1-Mo1-N2	83.43(16)
C5-C6	1.392(8)	O3-Mo1-N2	79.46(16)
C6-N1	1.398(7)	C1-O1-Mo1	114.8(3)
N1-C7	1.407(7)	O1-C1-C2	123.5(5)
C7-C12	1.409(7)	O1-C1-C6	115.7(5)
C7-C8	1.424(8)	C2-C1-C6	120.8(5)
C8-C9	1.370(8)	C3-C2-C1	117.2(5)
C9-C10	1.368(9)	C3-C2-C20	122.2(6)
C10-C11	1.370(8)	C1-C2-C20	120.5(6)
C11-C12	1.411(8)	C2-C3-C4	123.1(6)
C12-C13	1.454(7)	C5-C4-C3	118.6(6)
C13-N2	1.306(6)	C5-C4-C24	122.5(6)
C13-O2	1.350(6)	C3-C4-C24	118.9(6)
N2-C14	1.404(7)	C4-C5-C6	120.1(5)
C14-C15	1.374(7)	C5-C6-N1	127.3(5)
C14-C19	1.393(7)	C5-C6-C1	120.2(5)
C15-C16	1.377(8)	N1-C6-C1	112.5(5)
C16-C17	1.423(7)	C6-N1-C7	122.5(5)
C16-C28	1.523(7)	C6-N1-Mo1	111.0(3)
C17-C18	1.388(8)	C7-N1-Mo1	126.2(4)
C18-C19	1.382(7)	N1-C7-C12	121.8(5)
C18-C32	1.535(7)	N1-C7-C8	121.1(5)
C19-O2	1.395(6)	C12-C7-C8	117.1(5)
C20-C23	1.508(10)	C9-C8-C7	121.1(6)
C20-C21	1.515(9)	C10-C9-C8	121.5(6)
C20-C22	1.532(9)	C9-C10-C11	119.3(6)
C24-C26B	1.514(10)	C10-C11-C12	121.4(6)
C24-C25B	1.516(10)	C7-C12-C11	119.5(5)
C24-C25	1.517(9)	C7-C12-C13	121.7(5)
C24-C27	1.518(9)	C11-C12-C13	118.3(5)
C24-C26	1.519(9)	N2-C13-O2	113.9(4)
C24-C27B	1.525(10)	N2-C13-C12	127.9(5)
C28-C30	1.534(9)	O2-C13-C12	118.1(4)
C28-C31	1.537(9)	C13-N2-C14	106.0(4)
C28-C29	1.546(8)	C13-N2-Mo1	123.4(3)
C32-C33	1.512(8)	C14-N2-Mo1	130.0(3)
C32-C34	1.532(8)	C15-C14-C19	121.5(5)
C32-C35	1.551(9)	C15-C14-N2	131.0(5)
O3-C36	1.316(6)	C19-C14-N2	107.5(5)
N3-C41	1.312(7)	C14-C15-C16	117.5(5)
C36-C37	1.393(8)	C15-C16-C17	119.4(5)
C36-C41	1.427(7)	C15-C16-C28	122.6(5)
C37-C38	1.387(8)	C17-C16-C28	117.9(5)
C37-C42	1.543(8)	C18-C17-C16	124.3(5)
C38-C39	1.404(8)	C19-C18-C17	113.3(5)
C39-C40	1.367(8)	C19-C18-C32	122.1(5)
C39-C46	1.550(8)	C17-C18-C32	124.5(5)
C40-C41	1.403(7)	C18-C19-C14	123.8(5)
C42-C44	1.514(10)	C18-C19-O2	129.0(5)
C42-C45	1.525(10)	C14-C19-O2	107.2(5)
C42-C43	1.528(10)	C13-O2-C19	105.4(4)
C46-C48	1.510(8)	C23-C20-C21	111.4(7)
C46-C47B	1.514(10)	C23-C20-C22	107.9(7)
C46-C49B	1.515(10)	C21-C20-C22	107.0(6)
C46-C48B	1.518(10)	C23-C20-C2	109.7(6)
C46-C47	1.522(8)	C21-C20-C2	109.3(5)
C46-C49	1.525(9)	C22-C20-C2	111.5(6)

C40-C41-C36	120.6(6)	C26B-C24-C25B	110(2)
C44-C42-C45	108.3(7)	C25-C24-C27	109.6(12)
C44-C42-C43	108.0(6)	C25-C24-C26	105.4(12)
C45-C42-C43	109.6(6)	C27-C24-C26	110.8(14)
C44-C42-C37	112.8(6)	C26B-C24-C27B	104(2)
C45-C42-C37	108.6(6)	C25B-C24-C27B	114(3)
C43-C42-C37	109.5(6)	C26B-C24-C4	117.2(15)
C47B-C46-C49B	105(2)	C25B-C24-C4	108.1(16)
C47B-C46-C48B	118(2)	C25-C24-C4	109.4(10)
C49B-C46-C48B	105(2)	C27-C24-C4	114.6(9)
C48-C46-C47	109.0(9)	C26-C24-C4	106.5(9)
C48-C46-C49	107.6(11)	C27B-C24-C4	102.9(12)
C47-C46-C49	109.3(11)	C16-C28-C30	110.8(5)
C48-C46-C39	112.7(7)	C16-C28-C31	108.8(5)
C47B-C46-C39	112.7(14)	C30-C28-C31	109.7(6)
C49B-C46-C39	109.2(11)	C16-C28-C29	111.7(5)
C48B-C46-C39	106.7(12)	C30-C28-C29	108.6(6)
C47-C46-C39	109.1(7)	C31-C28-C29	107.2(5)
C49-C46-C39	109.1(9)	C33-C32-C34	108.7(5)
C40-C41-C36	120.6(6)	C33-C32-C18	110.6(5)
C44-C42-C45	108.3(7)	C34-C32-C18	111.1(5)
C44-C42-C43	108.0(6)	C33-C32-C35	110.5(5)
C45-C42-C43	109.6(6)	C34-C32-C35	108.7(5)
C44-C42-C37	112.8(6)	C18-C32-C35	107.3(5)
C45-C42-C37	108.6(6)	C36-O3-Mo1	115.3(3)
C43-C42-C37	109.5(6)	C41-N3-Mo1	118.3(3)
C47B-C46-C49B	105(2)	O3-C36-C37	126.2(5)
C47B-C46-C48B	118(2)	O3-C36-C41	113.6(5)
C49B-C46-C48B	105(2)	C37-C36-C41	120.2(5)
C48-C46-C47	109.0(9)	C38-C37-C36	116.7(5)
C48-C46-C49	107.6(11)	C38-C37-C42	122.3(6)
C47-C46-C49	109.3(11)	C36-C37-C42	121.0(5)
C48-C46-C39	112.7(7)	C37-C38-C39	124.0(6)
C47B-C46-C39	112.7(14)	C40-C39-C38	118.9(5)
C49B-C46-C39	109.2(11)	C40-C39-C46	121.9(5)
N3-C41-C40	125.3(5)	C38-C39-C46	119.2(5)
N3-C41-C36	114.2(5)	C39-C40-C41	119.4(5)

Symmetry transformations used to generate equivalent atoms

Crystal information of complex MoOL^{BIS}L^{SQ}

Symmetry transformations used to generate equivalent atoms:
The O4-Mo1-O3 angle of 75.54(15)° is the smallest, since it involves two donor groups of the **L^{SQ}** ligand. The valence angles between coordination bonds to Mo1 involving O1, N1 and N2 of **L^{BIS}** are much smaller than those expected for the octahedral sphere (Table 2), indicating the strain caused by its three-dentate coordination. The hydrophobic interactions between **L^{BIS}** and **L^{SQ}**, in particular those formed by *tBu* substituents at C2 and C16 (Figure 1) result in positioning of the latter in such a way, that the angles between bonds formed by **L^{SQ}** and **L^{BIS}** ligands range from O3-Mo1-N2 79.43(15) to O4-Mo1-N2 106.36(15)°, while the trans angle O4-Mo1-N1 is 157.65(18)°.
Conformation of the **L^{BIS}** ligand reflects the strain resulting from its three-dentate coordination. The C1-C6-N1-C7 and C6-N1-C7-C8 torsion angles are 153.6(5) and -24.0(9)°, while C7-C12-C13-N2 angle is 14.7(9)°. In the conformation of **L^{BIS}**, the dihedral angles between the phenolic and the central phenyl and benzoxazole rings are 43.6(3)° and 53.1(3)°, respectively. The central phenyl ring forms the dihedral angle of 15.9(3)° to the benzoxazole ring plane. The dihedral angles between the semiquinone ring and phenolate, central phenyl and benzoxazole rings are 55.9(3), 80.8(3) and 83.5(3)°, respectively.
The Conformation of the chelate ring Mo1-O1-C1-C6-N1 is an envelope on Mo1, Mo1-O3-C36-C41-O4 is twisted on O4-Mo1, Mo1-N1-C7-C12 ligands. -C13-N2 is an envelope on Mo1.
Some intra-molecular C-H···X interactions are found involving C-H groups of **L^{BIS}** and **L^{SQ}**, and O1, O2 of **L^{BIS}** and O3 of **L^{SQ}**. Also, analysis of the crystal packing also revealed the inter-molecular interactions C12···C48[1+x,y,z] of 3.451(15) and C13···C48[1+x,y,z] of 3.355(15) Å.

Table S2 Torsion angles [°] for MoOL^{BIS}L^{SQ}

Mo(1)-O(1)-C(1)-C(2)	-161.4(5)
Mo(1)-O(1)-C(1)-C(6)	17.6(6)
O(1)-C(1)-C(2)-C(3)	177.1(6)
C(6)-C(1)-C(2)-C(3)	-1.8(9)
O(1)-C(1)-C(2)-C(20)	-1.8(9)
C(6)-C(1)-C(2)-C(20)	179.3(6)
C(1)-C(2)-C(3)-C(4)	-0.6(10)
C(20)-C(2)-C(3)-C(4)	178.3(7)
C(2)-C(3)-C(4)-C(5)	1.2(11)
C(2)-C(3)-C(4)-C(24)	-178.5(7)
C(3)-C(4)-C(5)-C(6)	0.7(10)
C(24)-C(4)-C(5)-C(6)	-179.7(7)
C(4)-C(5)-C(6)-N(1)	178.9(6)
C(4)-C(5)-C(6)-C(1)	-3.0(9)
O(1)-C(1)-C(6)-C(5)	-175.4(5)
C(2)-C(1)-C(6)-C(5)	3.6(9)
O(1)-C(1)-C(6)-N(1)	3.0(7)
C(2)-C(1)-C(6)-N(1)	-178.0(5)
C(5)-C(6)-N(1)-C(7)	-27.8(9)
C(1)-C(6)-N(1)-C(7)	154.0(5)
C(5)-C(6)-N(1)-Mo(1)	157.4(5)
C(1)-C(6)-N(1)-Mo(1)	-20.8(6)
C(6)-N(1)-C(7)-C(12)	156.5(6)
Mo(1)-N(1)-C(7)-C(12)	-29.6(8)
C(6)-N(1)-C(7)-C(8)	-24.5(9)
Mo(1)-N(1)-C(7)-C(8)	149.4(5)
N(1)-C(7)-C(8)-C(9)	178.8(6)
C(12)-C(7)-C(8)-C(9)	-2.1(10)
C(7)-C(8)-C(9)-C(10)	0.5(11)
C(8)-C(9)-C(10)-C(11)	1.1(11)
C(9)-C(10)-C(11)-C(12)	-1.1(11)
N(1)-C(7)-C(12)-C(11)	-178.8(6)
C(8)-C(7)-C(12)-C(11)	2.1(9)
N(1)-C(7)-C(12)-C(13)	-7.2(9)
C(8)-C(7)-C(12)-C(13)	173.7(6)
C(10)-C(11)-C(12)-C(7)	-0.6(10)
C(10)-C(11)-C(12)-C(13)	-172.5(6)
C(7)-C(12)-C(13)-N(2)	15.1(9)
C(11)-C(12)-C(13)-N(2)	-173.2(6)
C(7)-C(12)-C(13)-O(2)	-160.9(5)
C(11)-C(12)-C(13)-O(2)	10.9(8)
O(2)-C(13)-N(2)-C(14)	1.1(6)
C(12)-C(13)-N(2)-C(14)	-175.0(6)
O(2)-C(13)-N(2)-Mo(1)	-171.0(3)
C(12)-C(13)-N(2)-Mo(1)	12.9(8)
C(13)-N(2)-C(14)-C(15)	179.3(6)
Mo(1)-N(2)-C(14)-C(15)	-9.4(9)
C(13)-N(2)-C(14)-C(19)	-1.2(6)
Mo(1)-N(2)-C(14)-C(19)	170.1(4)
C(19)-C(14)-C(15)-C(16)	0.0(9)
N(2)-C(14)-C(15)-C(16)	179.5(6)
C(14)-C(15)-C(16)-C(17)	-1.8(8)
C(14)-C(15)-C(16)-C(28)	-179.5(6)
C(15)-C(16)-C(17)-C(18)	2.4(9)
C(28)-C(16)-C(17)-C(18)	-179.8(5)
C(16)-C(17)-C(18)-C(19)	-0.9(9)
C(16)-C(17)-C(18)-C(32)	-179.8(5)
C(17)-C(18)-C(19)-C(14)	-1.0(8)
C(32)-C(18)-C(19)-C(14)	177.9(5)
C(17)-C(18)-C(19)-O(2)	-179.8(6)
C(32)-C(18)-C(19)-O(2)	-0.8(9)
C(15)-C(14)-C(19)-C(18)	1.6(9)
N(2)-C(14)-C(19)-C(18)	-178.0(5)
C(15)-C(14)-C(19)-O(2)	-179.5(5)
N(2)-C(14)-C(19)-O(2)	0.9(6)
N(2)-C(13)-O(2)-C(19)	-0.5(6)
C(12)-C(13)-O(2)-C(19)	176.0(5)
C(18)-C(19)-O(2)-C(13)	178.6(6)
C(14)-C(19)-O(2)-C(13)	-0.3(6)
C(3)-C(2)-C(20)-C(23)	123.7(7)

C(1)-C(2)-C(20)-C(23)	-57.4(8)
C(3)-C(2)-C(20)-C(21)	-113.9(7)
C(1)-C(2)-C(20)-C(21)	65.0(8)
C(3)-C(2)-C(20)-C(22)	4.2(10)
C(1)-C(2)-C(20)-C(22)	-176.9(6)
C(5)-C(4)-C(24)-C(26B)	152.5(19)
C(3)-C(4)-C(24)-C(26B)	-28(2)
C(5)-C(4)-C(24)-C(25B)	-82(2)
C(3)-C(4)-C(24)-C(25B)	98(2)
C(5)-C(4)-C(24)-C(25)	-123.3(11)
C(3)-C(4)-C(24)-C(25)	56.3(12)
C(5)-C(4)-C(24)-C(27)	0.3(15)
C(3)-C(4)-C(24)-C(27)	179.9(12)
C(5)-C(4)-C(24)-C(26)	123.2(11)
C(3)-C(4)-C(24)-C(26)	-57.1(12)
C(5)-C(4)-C(24)-C(27B)	39(2)
C(3)-C(4)-C(24)-C(27B)	-142(2)
C(15)-C(16)-C(28)-C(30)	-132.9(6)
C(17)-C(16)-C(28)-C(30)	49.3(8)
C(15)-C(16)-C(28)-C(31)	106.4(7)
C(17)-C(16)-C(28)-C(31)	-71.3(7)
C(15)-C(16)-C(28)-C(29)	-11.7(8)
C(17)-C(16)-C(28)-C(29)	170.5(5)
C(19)-C(18)-C(32)-C(33)	56.6(7)
C(17)-C(18)-C(32)-C(33)	-124.5(6)
C(19)-C(18)-C(32)-C(34)	177.4(5)
C(17)-C(18)-C(32)-C(34)	-3.7(8)
C(19)-C(18)-C(32)-C(35)	-63.9(7)
C(17)-C(18)-C(32)-C(35)	115.0(6)
Mo(1)-O(3)-C(36)-C(37)	-170.1(4)
Mo(1)-O(3)-C(36)-C(41)	9.0(6)
O(3)-C(36)-C(37)-C(38)	174.9(5)
O(3)-C(36)-C(37)-C(42)	-5.4(9)
C(41)-C(36)-C(37)-C(42)	175.6(5)
C(36)-C(37)-C(38)-C(39)	1.1(9)
C(42)-C(37)-C(38)-C(39)	-178.6(6)
C(37)-C(38)-C(39)-C(40)	2.6(10)
C(37)-C(38)-C(39)-C(46)	-176.0(6)
C(38)-C(39)-C(40)-C(41)	-3.2(8)
C(46)-C(39)-C(40)-C(41)	175.4(5)
Mo(1)-N(3)-C(41)-C(40)	162.9(4)
Mo(1)-N(3)-C(41)-C(36)	-17.4(6)
C(39)-C(40)-C(41)-N(3)	179.9(5)
C(39)-C(40)-C(41)-C(36)	0.2(8)
O(3)-C(36)-C(41)-N(3)	4.7(7)
C(37)-C(36)-C(41)-N(3)	-176.2(5)
O(3)-C(36)-C(41)-C(40)	-175.5(5)
C(37)-C(36)-C(41)-C(40)	3.6(8)
C(38)-C(37)-C(42)-C(44)	1.0(9)
C(36)-C(37)-C(42)-C(44)	-178.6(6)
C(36)-C(37)-C(42)-C(45)	61.4(8)
C(38)-C(37)-C(42)-C(43)	121.4(7)
C(36)-C(37)-C(42)-C(43)	-58.3(8)
C(40)-C(39)-C(46)-C(48)	-3.2(11)
C(38)-C(39)-C(46)-C(48)	175.4(9)
C(40)-C(39)-C(46)-C(47B)	154(2)
C(38)-C(39)-C(46)-C(47B)	-27(2)
C(40)-C(39)-C(46)-C(49B)	37.7(16)
C(38)-C(39)-C(46)-C(49B)	-143.7(15)
C(40)-C(39)-C(46)-C(48B)	-75(2)
C(38)-C(39)-C(46)-C(48B)	103(2)
C(40)-C(39)-C(46)-C(47)	-124.4(9)
C(38)-C(39)-C(46)-C(47)	54.2(10)
C(40)-C(39)-C(46)-C(49)	116.3(10)
C(38)-C(39)-C(46)-C(49)	-65.1(10)

Symmetry transformations used to generate equivalent atoms

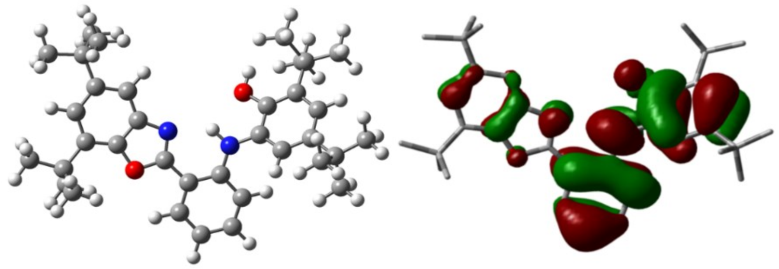

Figure S3 Optimized structure and HOMO (hydrogen atoms omitted for clarity) of compound H_2L^{BAP}

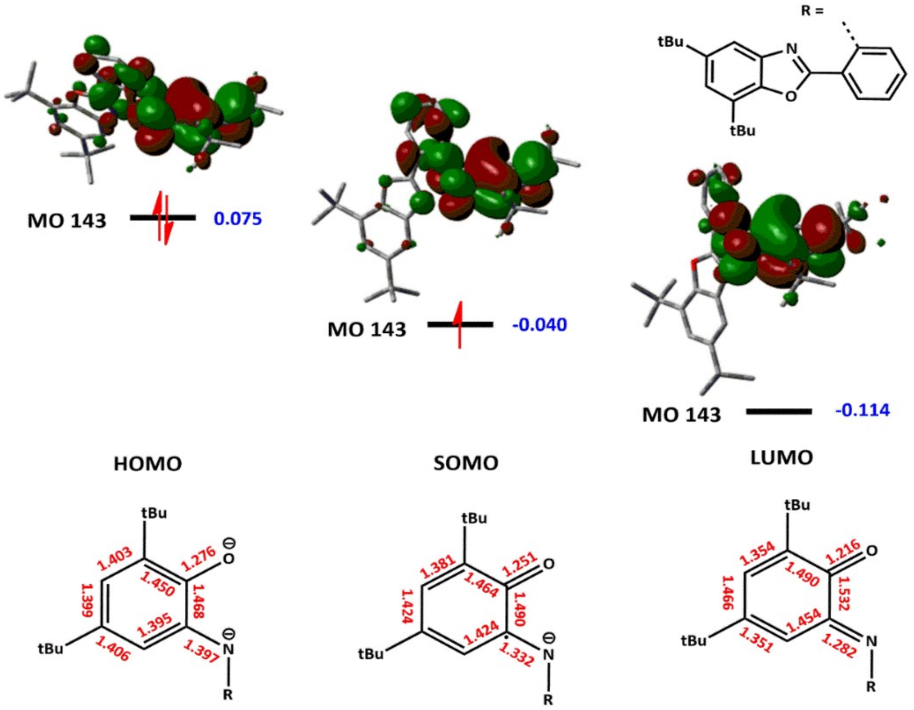

Figure S4 Selected bond lengths, MOs (hydrogen atoms omitted for clarity) and energies of optimized $(L^{BIS})^{n-}$ ligands (n = 2, 1, 0)

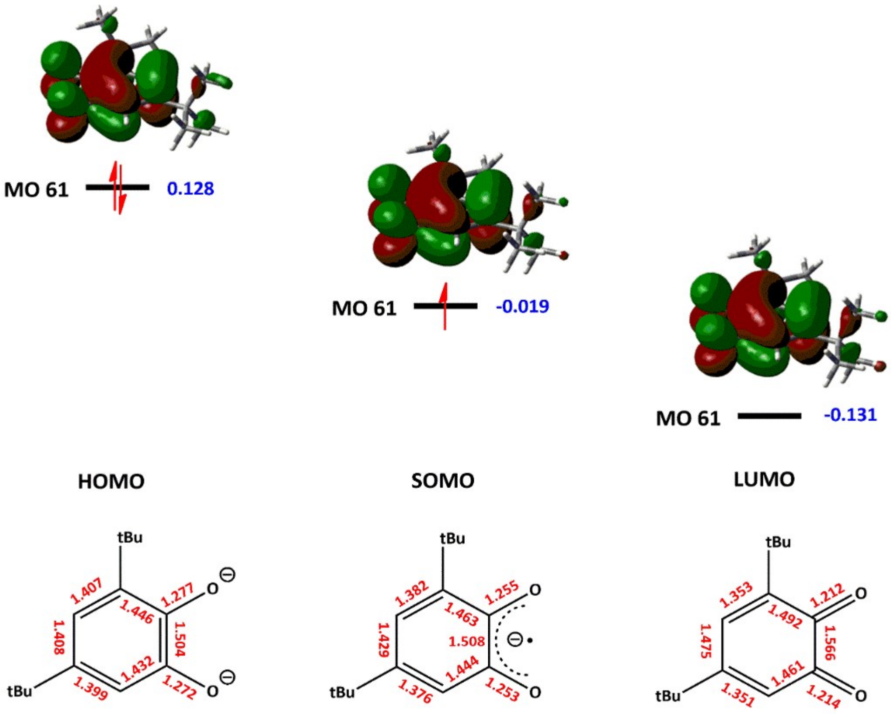

Figure S5 Selected bond lengths, MOs (hydrogen atoms omitted for clarity) and energies of optimized SQ^{n-} ligands (n = 2, 1, 0)

Table S3 Coordinates of the optimized compounds

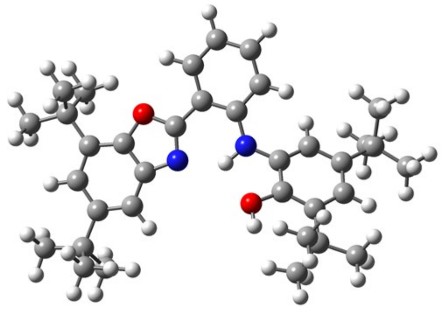

H_2L^{BAP}

O	1.94565092	1.61626676	-1.37627073
C	2.97559738	1.02154331	-0.68662447
C	4.24014477	1.59565125	-0.48150900
C	5.16878710	0.84617757	0.26095207
H	6.14650768	1.26811463	0.42558201
C	4.88578878	-0.40855021	0.80266967
C	3.61298923	-0.93912059	0.57760356
H	3.33259443	-1.89009488	1.01057817
C	2.66182293	-0.25467303	-0.17474145
N	1.35974130	-0.73814662	-0.39097836
C	0.96270855	-2.02908168	-0.65154026
C	1.88411772	-3.04668310	-0.98225298
H	2.93350008	-2.79907271	-1.05607148
C	1.46871110	-4.34029721	-1.24622653
H	2.21008710	-5.08829652	-1.50616174
C	0.11416750	-4.68562591	-1.19546263
H	-0.20807635	-5.69960585	-1.39736250
C	-0.81185697	-3.70110564	-0.90043367
H	-1.86672826	-3.94155221	-0.87694779
C	-0.42518095	-2.37511128	-0.63270855
C	-1.45106479	-1.38580191	-0.36200059
N	-1.31892371	-0.09985080	-0.19601271
C	-2.60739249	0.38218203	0.01650048
C	-3.06555112	1.67462094	0.24814193
H	-2.35873424	2.49355524	0.28474531
C	-4.43877988	1.86648407	0.42565489
C	-5.29809092	0.74950868	0.36380576
H	-6.35486360	0.91497227	0.50158491
C	-4.87576164	-0.56996215	0.13243529
C	-3.49956752	-0.69097959	-0.03580810
O	-2.75583152	-1.82656395	-0.27788296
C	-5.82703766	-1.77312777	0.06472072
C	-4.97232779	3.29186952	0.68057635
C	4.61258203	2.98623440	-1.05047641
C	5.90054001	-1.20266476	1.64751691
C	4.50643146	2.98978345	-2.59755367
H	5.19624285	2.25654710	-3.02300568
H	3.51247975	2.74521633	-2.97895911
H	4.77454502	3.97519469	-2.99033822
C	3.71334792	4.08918651	-0.43274933
H	2.64466097	3.97052616	-0.63332982
H	3.82632624	4.10455737	0.65412572
H	4.00316415	5.07075868	-0.81930375
C	6.06633029	3.37948497	-0.71547642
H	6.78699186	2.67190520	-1.13223075
H	6.28032470	4.36066280	-1.14768650
H	6.23473047	3.45071146	0.36178306
C	7.25369121	-0.47939230	1.77556040
H	7.93663938	-1.08435968	2.37842560
H	7.72546855	-0.32241528	0.80150802
H	7.15313928	0.49110956	2.26920640
C	6.15624131	-2.58044004	0.99334102
H	6.87536330	-3.15605866	1.58476434
H	5.24014714	-3.17135349	0.91986843
H	6.56189287	-2.46394705	-0.01566026

C	5.33296391	-1.41284918	3.07098976
H	5.14756470	-0.45324611	3.56155160
H	4.39068954	-1.96540307	3.05491003
H	6.04126771	-1.97849631	3.68473723
C	-5.71453452	-2.43935733	-1.32716641
H	-4.70033699	-2.78822041	-1.52705861
H	-6.38673293	-3.30109895	-1.38644907
H	-5.99425910	-1.73695172	-2.11739804
C	-5.44249762	-2.79486910	1.16107039
H	-4.41966212	-3.15357461	1.03748784
H	-5.52820687	-2.34769680	2.15544630
H	-6.11207618	-3.65964205	1.11974246
C	-7.29484739	-1.36455144	0.28338409
H	-7.64210202	-0.66296036	-0.48005753
H	-7.92952682	-2.25306530	0.22692135
H	-7.45094029	-0.91023893	1.26566085
C	-4.60700508	4.19819669	-0.51838324
H	-3.52686257	4.25204214	-0.67216623
H	-5.05683714	3.82221508	-1.44155961
H	-4.97224159	5.21648988	-0.35154387
C	-6.50150221	3.33279204	0.85749839
H	-7.02557057	2.97527089	-0.03309499
H	-6.83013145	2.73652483	1.71315344
H	-6.82053005	4.36365471	1.03398985
C	-4.32809668	3.86068443	1.96652086
H	-4.57319938	3.23981847	2.83272740
H	-3.23956650	3.90870019	1.88887770
H	-4.69430906	4.87406276	2.15923854
H	0.61646223	-0.04204248	-0.40692016
H	2.18121755	2.51420842	-1.61811668

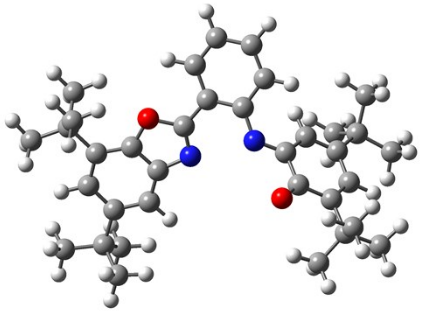

[L^{BIS}]$^-$

O	2.60527805	0.97067169	-2.46298326
C	3.14487401	0.64121845	-1.38384321
C	4.35487357	1.28972392	-0.87444974
C	4.93028441	0.83848509	0.29740332
H	5.82353154	1.33022265	0.65294241
C	4.40832939	-0.23596676	1.07266554
C	3.26293721	-0.86269438	0.62460155
H	2.81210098	-1.65939061	1.20631272
C	2.58515393	-0.47029627	-0.56459596
N	1.42596495	-0.99531402	-0.95853385
C	0.98445814	-2.23182723	-0.66577863
C	1.83589681	-3.37720536	-0.76096151
H	2.88530194	-3.20145698	-0.96457116
C	1.35021573	-4.66497840	-0.67356346
H	2.03544011	-5.50113205	-0.78260706
C	-0.01875010	-4.90563062	-0.46242827
H	-0.40112025	-5.91783849	-0.39569041
C	-0.87148233	-3.82253108	-0.33466020
H	-1.92685542	-3.98955917	-0.15599105
C	-0.40724980	-2.49530841	-0.40997789
C	-1.34825207	-1.41453257	-0.19709333
N	-1.15462494	-0.18464304	0.17199655
C	-2.41673341	0.38309850	0.24717099
C	-2.80646057	1.67470914	0.58869316
H	-2.04741511	2.40568657	0.83788674
C	-4.17101707	1.98330454	0.58772506

C	-5.09971171	0.98067487	0.24293560
H	-6.14877166	1.23271450	0.24485300
C	-4.74704631	-0.33539814	-0.10768486
C	-3.37744634	-0.57895416	-0.08660118
O	-2.70430735	-1.73699855	-0.37416674
C	-5.76715215	-1.41989307	-0.48450988
C	-4.61433521	3.41514824	0.95464816
C	4.95711555	2.46263439	-1.67200249
C	5.06176902	-0.68647973	2.39596561
C	5.37749418	1.97981365	-3.08224977
H	6.14604962	1.20308108	-3.00687869
H	4.51988380	1.57103393	-3.61502559
H	5.79451268	2.81372376	-3.66083536
C	3.91396722	3.59902672	-1.80809968
H	3.01847236	3.23643852	-2.31108198
H	3.63047748	3.97823636	-0.82072777
H	4.33448217	4.43288774	-2.38393597
C	6.20654416	3.06081856	-0.99501606
H	7.01313258	2.32740663	-0.89780427
H	6.58437031	3.88895474	-1.60377757
H	5.98290872	3.45653574	0.00063210
C	6.35073475	0.09197826	2.72257420
H	6.77473336	-0.27746933	3.66227923
H	7.10787484	-0.03275550	1.94318994
H	6.16013447	1.16199969	2.84272513
C	5.42901503	-2.18723815	2.31572925
H	5.88783483	-2.52469880	3.25310460
H	4.54894323	-2.80606719	2.12912244
H	6.13919667	-2.36658643	1.50302772
C	4.07129902	-0.47217466	3.56538715
H	3.80033790	0.58400312	3.65142169
H	3.14915438	-1.03746758	3.41544863
H	4.51666143	-0.79170179	4.51558230
C	-5.47834012	-1.92327532	-1.91875435
H	-4.47444373	-2.34168729	-2.00016110
H	-6.19756535	-2.70091556	-2.19849314
H	-5.56385892	-1.10532167	-2.63994139
C	-5.65083756	-2.60183159	0.50714247
H	-4.64878345	-3.03229281	0.49611950
H	-5.86917096	-2.27320627	1.52759651
H	-6.36433111	-3.39001616	0.24284311
C	-7.21525331	-0.89934124	-0.44073714
H	-7.37382948	-0.07691516	-1.14402209
H	-7.89986463	-1.70748065	-0.71556628
H	-7.49447887	-0.55398047	0.55884014
C	-4.00358322	4.41689613	-0.05326590
H	-2.91254851	4.37432501	-0.05328264
H	-4.34283412	4.19862258	-1.06983587
H	-4.30158731	5.44170835	0.19499137
C	-6.14416924	3.59287180	0.93070151
H	-6.56104910	3.40148778	-0.06202030
H	-6.64232565	2.93090385	1.64485598
H	-6.39710031	4.62248915	1.20180410
C	-4.12082760	3.76282117	2.37883486
H	-4.54417253	3.07349386	3.11521061
H	-3.03328857	3.70315831	2.45563986
H	-4.42136417	4.78082015	2.65063013

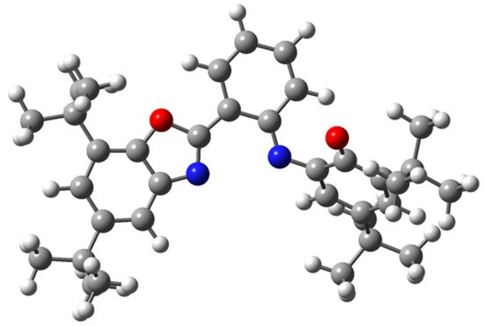

[L^BIS]^2-

O	-3.30827700	-1.66990700	1.56679700
C	-3.66943900	-0.70572900	0.81380800
C	-4.98897000	-0.10756500	0.88075900
C	-5.34038100	0.96145300	0.04288600
H	-6.33922700	1.37147100	0.12186000
C	-4.45585200	1.52337600	-0.88444500
C	-3.16835000	0.96129200	-0.94920800
H	-2.41972400	1.36833200	-1.62481400
C	-2.75492500	-0.12501700	-0.17750200
N	-1.41614300	-0.50760800	-0.29367800
C	-1.01921700	-1.72812400	-0.52139100
C	-1.92543900	-2.83595900	-0.82000800
H	-2.98296500	-2.62162400	-0.76097500
C	-1.49540700	-4.08344300	-1.15553800
H	-2.22691600	-4.86033200	-1.37314200
C	-0.10320700	-4.39453700	-1.21426200
H	0.23272700	-5.39412400	-1.47391800
C	0.80313000	-3.40445600	-0.92579300
H	1.86107100	-3.63474700	-0.96274000
C	0.41989800	-2.07535000	-0.57885100
C	1.45174400	-1.14147500	-0.30615300
N	1.45505500	0.13541100	0.02620400
C	2.76817800	0.50099500	0.15751800
C	3.34135200	1.73024500	0.49137700
H	2.68852100	2.57096600	0.69465100
C	4.73980400	1.83773300	0.55423000
C	5.53116800	0.70874300	0.28377000
H	6.60524300	0.80131200	0.33548300
C	4.99013200	-0.55560700	-0.05587300
C	3.60447400	-0.60215900	-0.10668500
O	2.79873900	-1.65613200	-0.40258000
C	5.85223200	-1.79501100	-0.34451200
C	5.36446200	3.20057700	0.92426300
C	-5.99381000	-0.67613900	1.90542600
C	-4.82402800	2.70061200	-1.80270800
C	-6.24760300	-2.17781100	1.62247700
H	-6.71565300	-2.30415600	0.63926200
H	-5.29653200	-2.71195300	1.63326500
H	-6.91930400	-2.61022800	2.37937900
C	-5.42202100	-0.52222800	3.33691400
H	-4.44665300	-1.00814500	3.38830200
H	-5.30272600	0.53903400	3.58337600
H	-6.09750500	-0.97344000	4.07939400
C	-7.36238300	0.03491500	1.87585000
H	-7.85034000	-0.05765000	0.89955400
H	-8.02380400	-0.41923800	2.62480900
H	-7.27432900	1.10089400	2.11077600
C	-6.27129400	3.18799400	-1.59524800
H	-6.48597100	4.02427100	-2.27252700
H	-6.99408500	2.39270800	-1.80037700
H	-6.43414400	3.53347200	-0.57006000
C	-4.68181900	2.28580800	-3.28880000
H	-4.91617000	3.12376100	-3.96184100
H	-3.66659700	1.94663700	-3.50543800
H	-5.36009700	1.45793400	-3.51717800
C	-3.88508700	3.90374300	-1.53550300
H	-3.99330400	4.24560900	-0.50164500
H	-2.83889100	3.62599100	-1.67972200
H	-4.11362500	4.74539800	-2.20592400
C	5.49814700	-2.91555700	0.66193800
H	4.44178500	-3.17951300	0.60055100
H	6.09249900	-3.81525000	0.45947700
H	5.70697800	-2.59124000	1.68616000
C	5.57477900	-2.29589000	-1.78199400
H	4.52003100	-2.53855200	-1.91650300
H	5.84249400	-1.52747800	-2.51412800
H	6.16695800	-3.19424000	-1.99652300
C	7.35960500	-1.50344300	-0.22358500
H	7.62811700	-1.16918000	0.78299300
H	7.92670600	-2.41642700	-0.43539600
H	7.68250200	-0.73689000	-0.93445500

C	4.88734400	3.62752000	2.33263200
H	3.79952000	3.70663000	2.37880300
H	5.19636600	2.89251200	3.08167100
H	5.31256600	4.60085400	2.60800000
C	6.90463200	3.17141700	0.94044400
H	7.28861100	2.45621100	1.67348200
H	7.31365100	2.90664000	-0.03885300
H	7.28927700	4.16210100	1.20700200
C	4.92661400	4.27222800	-0.10190100
H	5.26234300	4.00092800	-1.10717600
H	3.84024800	4.37468600	-0.13423500
H	5.35348900	5.25083300	0.15190000

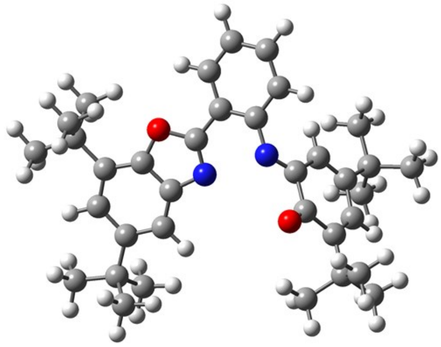

[L^BIS]

O	2.66143724	0.73221373	-2.66077102
C	2.99704204	0.48735702	-1.51784316
C	3.94390774	1.34032439	-0.74492717
C	4.35894741	0.87923053	0.45882965
H	5.06461396	1.47628750	1.01991617
C	3.93172473	-0.37230071	1.09263138
C	3.03434202	-1.15951620	0.45913792
H	2.66396018	-2.07136112	0.90531228
C	2.46893473	-0.77154690	-0.82325638
N	1.50820193	-1.37335206	-1.42184937
C	0.99614575	-2.60360150	-1.02590924
C	1.77597190	-3.76061291	-1.20190645
H	2.79456818	-3.64609235	-1.55322376
C	1.24360663	-5.02419604	-0.98436650
H	1.86489460	-5.89929305	-1.13956709
C	-0.08326618	-5.16694741	-0.57613749
H	-0.50368197	-6.15073004	-0.40576141
C	-0.86395250	-4.03506520	-0.38820730
H	-1.89150049	-4.13436653	-0.06299763
C	-0.35042507	-2.74675579	-0.60612575
C	-1.19518311	-1.58245812	-0.37255071
N	-0.85258591	-0.34414645	-0.20447841
C	-2.05037301	0.33914718	-0.01318204
C	-2.29187471	1.69153080	0.20767381
H	-1.45992924	2.38346375	0.24104484
C	-3.61522838	2.10766963	0.37233792
C	-4.64675105	1.14579383	0.31115866
H	-5.66273056	1.48411425	0.44031748
C	-4.44665516	-0.22609797	0.09117033
C	-3.10732489	-0.57332719	-0.06652926
O	-2.55715754	-1.81446343	-0.29788123
C	-5.58185142	-1.25822118	0.03019488
C	-3.90939484	3.60389740	0.60882528
C	4.42800678	2.66477340	-1.35216626
C	4.51674516	-0.70414598	2.47127124
C	5.22413419	2.38656840	-2.65196960
H	6.09332007	1.75402239	-2.44706331
H	4.60503560	1.89509536	-3.40059620
H	5.58802311	3.33070624	-3.06892676
C	3.21258136	3.57086978	-1.66810310
H	2.54184715	3.10409019	-2.38782122
H	2.64752325	3.79414586	-0.75807038
H	3.56154059	4.51932582	-2.08764713

C	5.35125191	3.43244736	-0.38737525
H	6.26532723	2.87618833	-0.15946340
H	5.65227280	4.37426989	-0.85304937
H	4.84972290	3.67732709	0.55369903
C	6.05737211	-0.80878961	2.36051009
H	6.48573300	-1.04007204	3.34016617
H	6.34529422	-1.60451125	1.66817930
H	6.51382855	0.12093301	2.01329985
C	3.98334262	-2.03675043	3.02501077
H	4.43309034	-2.23173353	4.00205491
H	2.89875409	-2.01690254	3.15919493
H	4.23362591	-2.87750582	2.37265474
C	4.14500821	0.41839481	3.47047605
H	4.54377066	1.39033514	3.17111957
H	3.06001038	0.51365417	3.56351915
H	4.55207867	0.18574789	4.45887486
C	-5.57015200	-1.95603110	-1.35068274
H	-4.62518032	-2.46878406	-1.53565729
H	-6.37413359	-2.69685397	-1.40327374
H	-5.72566947	-1.23040393	-2.15386828
C	-5.37641794	-2.31207026	1.14433546
H	-4.42468958	-2.83399603	1.03365215
H	-5.39423137	-1.84200981	2.13166064
H	-6.17747799	-3.05692690	1.10883279
C	-6.96409113	-0.61181994	0.23044086
H	-7.18587006	0.12632620	-0.54510664
H	-7.73562283	-1.38488393	0.17985522
H	-7.05003211	-0.12389837	1.20523088
C	-3.41088842	4.42142296	-0.60584460
H	-2.33742983	4.29769190	-0.76484393
H	-3.92083728	4.10894648	-1.52132022
H	-3.60676389	5.48765311	-0.45356760
C	-5.41028015	3.89485187	0.79386617
H	-5.99251535	3.61983191	-0.08981441
H	-5.82530365	3.36843646	1.65783843
H	-5.55503664	4.96570214	0.96104025
C	-3.17248717	4.07995694	1.88238627
H	-3.50837492	3.51964858	2.75947250
H	-2.09117887	3.95101729	1.79811570
H	-3.36900408	5.14196030	2.06105804

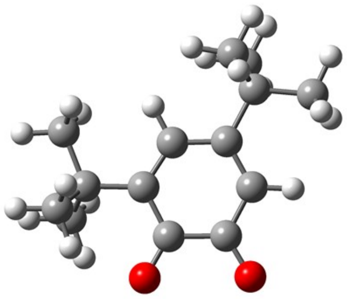

SQ^-

O	-2.39519574	1.87581672	-0.00027606
C	-1.26362746	1.33288691	-0.00010270
C	-1.08138639	-0.11859610	0.00001491
C	0.19576034	-0.64756076	0.00003581
H	0.31395954	-1.72407182	0.00017762
C	1.38495756	0.14511707	-0.00006452
C	1.25580679	1.51523284	-0.00024154
H	2.11511075	2.17603046	-0.00029972
C	-0.02159571	2.18763308	-0.00040776
O	-0.09904972	3.43835345	-0.00035453
C	-2.33079811	-1.02202261	0.00013795
C	2.74989860	-0.56902523	0.00007290
C	3.92998214	0.42062512	0.00030896
H	3.91427496	1.06411597	-0.88324300
H	3.91408979	1.06391837	0.88400044
H	4.87705192	-0.13065783	0.00034895
C	2.88032554	-1.45808800	1.26021178

H	2.09118306	-2.21224584	1.30400652
H	3.84676485	-1.97763558	1.27119261
H	2.80756871	-0.84966574	2.16631378
C	2.88070050	-1.45791883	-1.26014754
H	3.84716228	-1.97742863	-1.27093122
H	2.09160269	-2.21210151	-1.30426173
H	2.80817239	-0.84937905	-2.16618901
C	-1.98393605	-2.52417319	0.00019893
H	-2.91042343	-3.10906961	0.00030786
H	-1.41047025	-2.81263995	-0.88644453
H	-1.41032719	-2.81253405	0.88678441
C	-3.18189583	-0.74218927	-1.26286806
H	-3.47485073	0.30658907	-1.29535488
H	-2.60936507	-0.97382027	-2.16745633
H	-4.08428614	-1.36762336	-1.26255364
C	-3.18174494	-0.74205349	1.26321528
H	-2.60910417	-0.97358200	2.16776010
H	-3.47470014	0.30672704	1.29562193
H	-4.08413214	-1.36749233	1.26307824

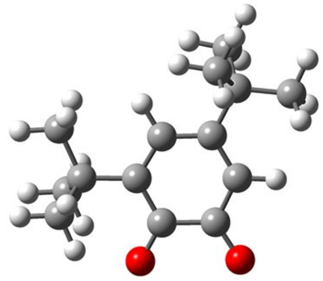

SQ^{2-}

O	-2.44051000	1.84673100	0.00134100
C	-1.28048000	1.31322500	0.00213300
C	-1.07683700	-0.11867400	-0.00224100
C	0.21932100	-0.66538600	0.00711700
H	0.33672900	-1.74315800	0.00921300
C	1.38095300	0.13015000	0.00084600
C	1.21831900	1.52007000	-0.00672900
H	2.07284600	2.19019200	-0.00889700
C	-0.05204900	2.18149800	0.00234100
O	-0.14492700	3.44954800	-0.00139300
C	-2.32195500	-1.03320100	-0.00072900
C	2.76587700	-0.55103600	-0.00245200
C	3.92326600	0.46621500	-0.04198300
H	3.87371700	1.09142900	-0.93866000
H	3.89714900	1.13048600	0.82662800
H	4.88798500	-0.05596600	-0.03826300
C	2.94808000	-1.40924900	1.27779900
H	2.19633300	-2.20024400	1.33621600
H	3.94404900	-1.88345000	1.31315500
H	2.83631800	-0.78443900	2.16911400
C	2.91247100	-1.47130600	-1.23855600
H	3.89301200	-1.96159600	-1.25744100
H	2.14397900	-2.24921100	-1.24339200
H	2.79907700	-0.88724200	-2.15809800
C	-1.97916400	-2.53724400	-0.01140000
H	-2.90833600	-3.12324100	-0.00729800
H	-1.40995600	-2.81875200	-0.90415900
H	-1.39494700	-2.83065700	0.86633100
C	-3.18735400	-0.75084400	-1.25526500
H	-3.44218900	0.30955500	-1.28482600
H	-2.62803900	-1.00729200	-2.16242600
H	-4.11004100	-1.35372600	-1.24048700
C	-3.16912900	-0.75988000	1.26745600
H	-2.59863100	-1.02536400	2.16518800
H	-3.41806600	0.30216400	1.30763600
H	-4.09542000	-1.35573900	1.26086500

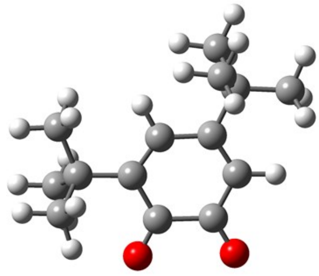

SQ

O	-2.31261439	1.93502369	-0.00030962
C	-1.24716675	1.35756320	-0.00016229
C	-1.10003518	-0.12692049	-0.00000590
C	0.16037651	-0.61790034	0.00004679
H	0.28885706	-1.69132382	0.00013373
C	1.40512388	0.17326049	-0.00000071
C	1.34267480	1.52255337	-0.00006182
H	2.22045063	2.15478056	-0.00007434
C	0.05956383	2.22038299	-0.00008302
O	-0.03544990	3.43063152	-0.00005436
C	-2.35516351	-1.00949413	0.00005129
C	2.72578623	-0.60427959	0.00004970
C	3.94926645	0.32862913	0.00006371
H	3.97275492	0.96813094	-0.88600880
H	3.97272519	0.96814438	0.88612711
H	4.86386166	-0.26959919	0.00008452
C	2.79359777	-1.49389368	1.26588416
H	1.97842147	-2.21930955	1.31108270
H	3.73305808	-2.05366247	1.27327650
H	2.75457824	-0.88532852	2.17318754
C	2.79366819	-1.49394378	-1.26574636
H	3.73313735	-2.05369861	-1.27307345
H	1.97850583	-2.21937413	-1.31095388
H	2.75468155	-0.88541531	-2.17307581
C	-2.00170648	-2.50828005	0.00012722
H	-2.92470420	-3.09326531	0.00017824
H	-1.43225719	-2.79793651	-0.88810412
H	-1.43221936	-2.79783578	0.88836722
C	-3.19728547	-0.71692363	-1.26677991
H	-3.53434202	0.31822731	-1.29363489
H	-2.62009267	-0.91993720	-2.17387912
H	-4.07858747	-1.36518149	-1.27901834
C	-3.19726274	-0.71679451	1.26686795
H	-2.62005061	-0.91970791	2.17397747
H	-3.53433049	0.31835538	1.29362188
H	-4.07855888	-1.36505848	1.27919290

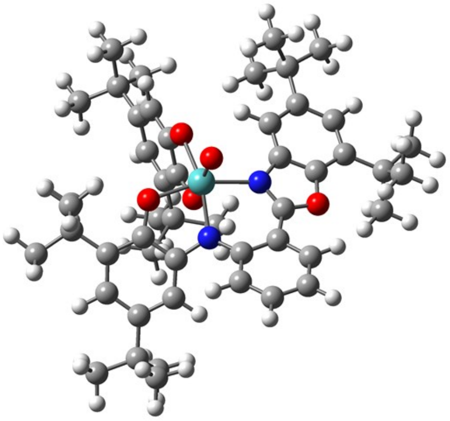

[MoO(L^{BIS})(SQ)] (S = 1)

Mo	0.41281587	-0.38180539	-1.63721253
O	0.10904445	-0.91847587	-3.22372973
O	2.12905552	0.64337890	-1.68871383
C	3.14824161	0.14998243	-0.96642917

C	4.35117202	0.84807176	-0.75439375
C	5.28460976	0.24644103	0.09981191
H	6.20951152	0.76944617	0.28437793
C	5.07233816	-0.98191614	0.74257538
C	3.88281154	-1.66533504	0.47603005
H	3.67587973	-2.61177910	0.95761299
C	2.94263763	-1.13342751	-0.40493746
N	1.71192812	-1.72182323	-0.77719510
C	1.50774314	-3.09353096	-0.72599690
C	2.56914749	-3.99951002	-0.95196042
H	3.54219918	-3.59838596	-1.19731974
C	2.38542465	-5.36815700	-0.89593519
H	3.22611088	-6.02386492	-1.09446932
C	1.12738414	-5.90929620	-0.60411337
H	0.98320398	-6.98141782	-0.55405915
C	0.06137539	-5.05269147	-0.41405346
H	-0.92484762	-5.45364691	-0.22207116
C	0.21679928	-3.65255674	-0.48932670
C	-0.97203471	-2.84155730	-0.35000411
N	-1.18967007	-1.60079295	-0.73145626
C	-2.53992814	-1.33160666	-0.46112577
C	-3.32846486	-0.21584610	-0.71155738
H	-2.89866538	0.65210295	-1.19030860
C	-4.67733674	-0.27904155	-0.34804332
C	-5.17400927	-1.45330015	0.25461119
H	-6.21550567	-1.47861581	0.53315170
C	-4.41054560	-2.60205505	0.51000161
C	-3.08335950	-2.47696789	0.11343702
O	-2.07585781	-3.42307921	0.18126615
O	0.22122929	0.57486606	0.45589040
C	-0.21961299	1.77445158	0.47678791
C	-0.27702562	2.61518042	1.64557280
C	-0.77710221	3.88627186	1.46573781
H	-0.82756492	4.54344027	2.32200821
C	-1.23708085	4.41241988	0.21240429
C	-1.20153732	3.60187857	-0.90636228
H	-1.53151146	3.93627132	-1.87999698
C	-0.70305667	2.28903268	-0.80482226
O	-0.65496146	1.47310375	-1.80794692
C	-4.96849275	-3.87949820	1.15323081
C	-5.58387727	0.93828818	-0.62296224
C	4.60800110	2.21001894	-1.42642569
C	6.09033282	-1.59010315	1.72663849
C	0.20412760	2.08756795	3.00587399
C	-1.74383710	5.86129792	0.16695753
C	-2.20529952	6.27500589	-1.24181790
H	-1.39217355	6.21587260	-1.96979515
H	-3.03090886	5.65343937	-1.59847052
H	-2.55609283	7.31028852	-1.22229431
C	-2.94250413	6.02093524	1.13337425
H	-2.67118539	5.79449225	2.16675049
H	-3.31075257	7.05099511	1.10489973
H	-3.76415976	5.35829917	0.84868161
C	-0.60446161	6.81546286	0.60104498
H	-0.95357967	7.85217851	0.57215864
H	-0.25984644	6.60904738	1.61679357
H	0.25469674	6.72687240	-0.06905816
C	0.03654236	3.13436806	4.12241106
H	0.38837563	2.71126695	5.06696981
H	0.62260245	4.03762005	3.93010386
H	-1.00952235	3.42287491	4.26174749
C	1.70437191	1.71617475	2.92008198
H	1.88634701	0.95243252	2.16518120
H	2.30689826	2.59525230	2.67350151
H	2.04676105	1.33388022	3.88704995
C	-0.61932491	0.83692264	3.39885384
H	-1.68323134	1.08085728	3.47643474
H	-0.49883560	0.03940657	2.66688085
H	-0.28652527	0.46511520	4.37304090
C	4.51211770	2.05613925	-2.96366487
H	5.26165139	1.34724046	-3.32815467

H	3.52718711	1.70218372	-3.26661434
H	4.69657050	3.02046655	-3.44836800
C	3.56431863	3.24503438	-0.94306536
H	2.55152443	2.93239959	-1.19354534
H	3.62747431	3.38162997	0.14059277
H	3.75337695	4.21390575	-1.41693431
C	6.00695470	2.76425091	-1.09593604
H	6.80385097	2.09403919	-1.43073533
H	6.14449575	3.72018499	-1.60889460
H	6.13628265	2.94565839	-0.02508123
C	7.35895801	-0.72872351	1.86943129
H	8.04688267	-1.20472568	2.57394634
H	7.88580079	-0.61832219	0.91756393
H	7.13315369	0.26957250	2.25345404
C	6.52032833	-2.99226691	1.23550839
H	7.24794266	-3.43178599	1.92536501
H	5.67135345	-3.67700030	1.16961836
H	6.98240474	-2.93395029	0.24595199
C	5.43978765	-1.71908876	3.12434188
H	5.13428256	-0.73941562	3.50190133
H	4.55379737	-2.35799254	3.10133251
H	6.14811532	-2.15418070	3.83700000
C	-4.82834818	-5.05890175	0.16147933
H	-3.78664554	-5.24341535	-0.10525348
H	-5.22782752	-5.97368829	0.60950527
H	-5.38471559	-4.86176968	-0.75889831
C	-4.18048072	-4.19041179	2.44803692
H	-3.11848415	-4.34422467	2.25120464
H	-4.27809283	-3.37341544	3.16825852
H	-4.57192957	-5.09994205	2.91332830
C	-6.45616808	-3.73593445	1.52034597
H	-7.08006223	-3.55433692	0.64109543
H	-6.80365115	-4.66293608	1.98378069
H	-6.62412534	-2.92696873	2.23641535
C	-5.56656296	1.25766129	-2.13640855
H	-4.56098088	1.48790441	-2.49434616
H	-5.94251493	0.41136919	-2.71815460
H	-6.20251665	2.12304880	-2.34670547
C	-7.04588500	0.69755257	-0.20407354
H	-7.49479897	-0.13943704	-0.74608487
H	-7.13908756	0.50506588	0.86839922
H	-7.63809663	1.58864326	-0.42835244
C	-5.05341723	2.15783880	0.16548648
H	-5.06258204	1.96138565	1.24134423
H	-4.03086783	2.41334925	-0.11930575
H	-5.68362824	3.03235599	-0.02468254

[MoO(L^{BIS})(SQ)] (S = 0)

Mo	0.37856500	-0.34087700	-1.59548800
O	-0.03837900	-0.84870500	-3.17136300
O	2.16508600	0.56302900	-1.68589200
C	3.12966800	0.06004800	-0.90395000
C	4.35928600	0.70194200	-0.66335600
C	5.21832100	0.07992400	0.25144500
H	6.15460400	0.56754200	0.47193100
C	4.92382900	-1.12304600	0.91376900
C	3.72112400	-1.76060500	0.60565600
H	3.44763200	-2.69009600	1.08816200
C	2.84779500	-1.19968400	-0.32496500
N	1.63274400	-1.75552300	-0.77527600
C	1.43949000	-3.12922900	-0.82365400
C	2.51504300	-4.01468200	-1.06201200
H	3.49667500	-3.59720000	-1.23703300
C	2.33042300	-5.38333700	-1.10379400
H	3.17856500	-6.02613600	-1.31195300
C	1.06161500	-5.94246500	-0.89827100
H	0.92005700	-7.01573700	-0.92794200
C	-0.01330100	-5.10379600	-0.68425000
H	-1.00478300	-5.51515000	-0.54764400

C	0.14521400	-3.70259500	-0.66126200
C	-1.02426500	-2.88572700	-0.46200500
N	-1.19045700	-1.60821000	-0.72706500
C	-2.51963900	-1.30113200	-0.40394700
C	-3.25059900	-0.12804000	-0.52938600
H	-2.78068400	0.76155800	-0.92261300
C	-4.59606200	-0.15916200	-0.15054800
C	-5.14439500	-1.36322500	0.33821300
H	-6.18283800	-1.36531900	0.62914400
C	-4.43829800	-2.56909800	0.46579800
C	-3.10988400	-2.47072700	0.06639200
O	-2.14613200	-3.46554700	0.02927300
O	0.20037400	0.57828100	0.37594300
C	-0.19626200	1.81845800	0.39462000
C	-0.30315500	2.65347100	1.54435100
C	-0.70221000	3.96273800	1.31353700
H	-0.76432100	4.62855100	2.16315400
C	-1.02990700	4.50557100	0.03774800
C	-0.96011900	3.67374600	-1.06560800
H	-1.20008900	4.00293100	-2.06712400
C	-0.55487900	2.34036100	-0.88869400
O	-0.51460800	1.45527500	-1.86079800
C	-5.05688000	-3.87635600	0.98063400
C	-5.43966400	1.12490600	-0.28432400
C	4.71383700	2.03070100	-1.35563600
C	5.86657300	-1.74617000	1.96156500
C	0.04946800	2.11606000	2.94060000
C	-1.43396300	5.98642900	-0.06229800
C	-1.75969900	6.40223200	-1.50837000
H	-0.89896700	6.27810400	-2.17089100
H	-2.59338100	5.82634700	-1.91943300
H	-2.04458900	7.45768100	-1.53112600
C	-2.68668600	6.24920000	0.80629300
H	-2.51031700	6.01868400	1.85944600
H	-2.97794000	7.30239000	0.74130800
H	-3.53009900	5.64160800	0.46715600
C	-0.26956100	6.87332000	0.44010300
H	-0.53899600	7.93155100	0.36332700
H	-0.02183600	6.66854600	1.48410100
H	0.63145400	6.70719000	-0.15645400
C	-0.16131700	3.17210300	4.04079100
H	0.08832600	2.73516100	5.01159500
H	0.48232000	4.04517600	3.90045600
H	-1.19979500	3.51258500	4.09005000
C	1.53588000	1.68669100	2.96760600
H	1.74044500	0.91052900	2.23068700
H	2.18760600	2.54014200	2.75861800
H	1.79471600	1.29922200	3.95847500
C	-0.84697200	0.89836700	3.26925600
H	-1.90255600	1.18607600	3.28559900
H	-0.71610700	0.10492900	2.53410800
H	-0.58999900	0.50164100	4.25673600
C	4.64875100	1.84982800	-2.89130400
H	5.36611300	1.09280900	-3.22247100
H	3.65354300	1.54655700	-3.21511500
H	4.90019800	2.79235100	-3.38823700
C	3.71979100	3.13270600	-0.91912500
H	2.69526100	2.87369400	-1.18259800
H	3.76796400	3.29122600	0.16221900
H	3.97237100	4.07793300	-1.41063000
C	6.13409400	2.50805600	-0.99842300
H	6.89729800	1.78061300	-1.28968800
H	6.34451200	3.43822200	-1.53330700
H	6.24328600	2.71425800	0.07014200
C	7.16281200	-0.93500300	2.14414900
H	7.79682900	-1.42251900	2.88991700
H	7.73668300	-0.86888600	1.21565100
H	6.96265900	0.08042100	2.49623400
C	6.25829000	-3.17806300	1.52751600
H	6.92916300	-3.63002300	2.26507600
H	5.38478400	-3.82781900	1.43359600

H	6.77326000	-3.16597600	0.56264100

Table S5 Experimental and selected list of calculated absorption properties of the $MoOL^{BIS}L^{SQ}$ complex

Exp. λ_{max}/nm	Tr[a]	Composition	Energy/eV (nm)	Oscillator strength	Assignment
450-542	5	HOMO→LUMO+2 (75%)<br>HOMO→LUMO+3 (13%)	2.32 (534)	0.03262	LMCT
	8	HOMO-3→LUMO (43%)<br>HOMO-1→LUMO+1 (23%)<br>HOMO-1→LUMO+2 (10%)	2.59 (479)	0.1213	LMCT
	9	HOMO→LUMO+2 (13%)<br>HOMO→LUMO+3 (72%)	2.71 (457)	0.1095	LMCT

C	5.14364800	-1.80862700	3.32774800
H	4.85808400	-0.80743200	3.66163900
H	4.23554900	-2.41421600	3.27775300
H	5.79817600	-2.24871500	4.08714500
C	-4.98035600	-4.95202700	-0.12905800
H	-3.94996000	-5.16128800	-0.42067900
H	-5.42639600	-5.88656100	0.22411100
H	-5.52707000	-4.63157500	-1.02011300
C	-4.27962400	-4.35818300	2.22861000
H	-3.22811300	-4.54554000	2.00568500
H	-4.33031900	-3.61504900	3.02912100
H	-4.71487400	-5.28926400	2.60391400
C	-6.53389100	-3.69725800	1.37410300
H	-7.15018500	-3.39202800	0.52419300
H	-6.92714600	-4.64917600	1.74011700
H	-6.65730400	-2.96121300	2.17304100
C	-5.43000800	1.59128000	-1.75915900
H	-4.41997400	1.80784300	-2.11289300
H	-5.85724700	0.82649400	-2.41382200
H	-6.02464900	2.50324700	-1.86937100
C	-6.90508900	0.91969800	0.14103200
H	-7.40884400	0.16629400	-0.47104600
H	-6.98987500	0.62589800	1.19092700
H	-7.45128200	1.85877400	0.01973800
C	-4.83061600	2.23100800	0.60824100
H	-4.83514600	1.93061400	1.65984900
H	-3.80021500	2.46014300	0.32987600
H	-5.41462800	3.15202300	0.51687600

Table S4 Comparison of experimental and theoretical structural parameters of complex $MoOL^{BIS}L^{SQ}$

Structural parameters (Å and º)	Experimental (X-ray)	Theoretical (DFT)<br>S = 1	Theoretical (DFT)<br>S = 0
Mo1-O1	1.955(4)	1.992	1.995
Mo1-O3	2.091(4)	2.305	2.169
Mo1-O4	1.993(4)	2.136	2.017
Mo1-O5	1.686(4)	1.700	1.705
Mo1-N1	2.037(4)	2.053	2.058
Mo1-N2	2.148(4)	2.207	2.196
O5-Mo1-O1	108.2(2)	106.9	108.1
O5-Mo1-O4	92.1(2)	96.0	91.3
O5-Mo1-N1	108.4(2)	107.5	108.4
O5-Mo1-O3	159.8(2)	164.2	159.7
O5-Mo1-N2	89.0(2)	94.5	91.1

344-380	13	HOMO-6→LUMO (13%)<br>HOMO-2→LUMO+1 (46%)<br>HOMO-1→LUMO+2 (24%)	3.11 (398)	0.0665	LMCT
	15	HOMO-2→LUMO+2 (86%)<br>HOMO-3→LUMO+1 (15%)	3.29 (377)	0.0461	LMCT
	17	HOMO-1→LUMO+2 (12%)<br>HOMO-1→LUMO+3 (54%)	3.45 (359)	0.0504	LMCT
296	25	HOMO-4→LUMO+2 (70%)	3.95 (313)	0.1889	LLCT
	32	HOMO-4→LUMO+3 (81%)	4.24 (292)	0.2419	LLCT
246	45	HOMO-6→LUMO+3 (25%)<br>HOMO-2→LUMO+4 (30%)	4.72 (263)	0.1471	LLCT
	47	HOMO-8→LUMO+1 (63%)	4.79 (257)	0.0681	LLCT

[a]Tr = transition number as obtained in the TD-DFT calculation output.

Table S6 Energies (eV) and composition (%) of some frontier molecular orbitals of $MoOL^{BIS}L^{SQ}$ complex

Orbital	Energy	Mo	O	L^{BIS}	L^{SQ}
LUMO+4	-0.64	6	0	93	1
LUMO+3	-1.68	45	13	39	4
LUMO+2	-2	36	11	49	4
LUMO+1	-2.31	48	18	31	4
LUMO	-3.31	41	1	23	35
HOMO	-5.07	4	1	50	45
HOMO-1	-5.89	25	1	34	40
HOMO-2	-5.98	1	2	96	1
HOMO-3	-6.39	9	0	8	83
HOMO-4	-6.51	0	0	99	0
HOMO-6	-7.07	1	1	96	3
HOMO-8	-7.74	0	0	99	1

Table S7 Molecular orbitals corresponding to TD-DFT excitations for the $MoOL^{BIS}L^{SQ}$ complex

**HOMO** 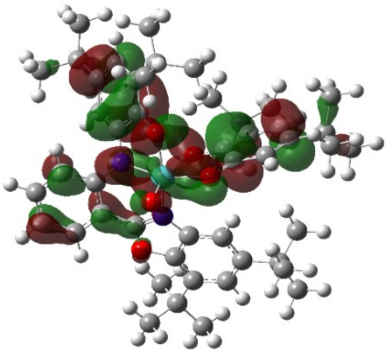	**HOMO-1** 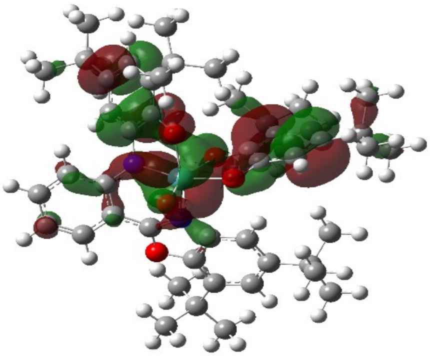
**HOMO-2** 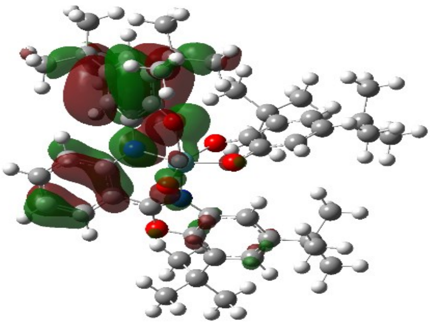	**HOMO-3** 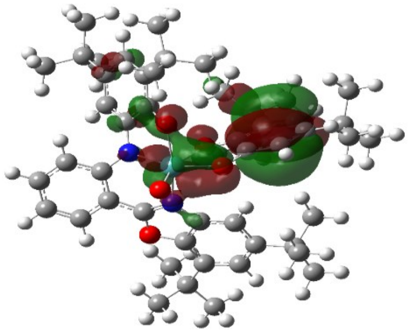
**HOMO-4** 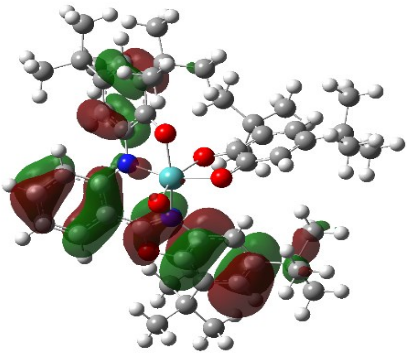	**HOMO-6** 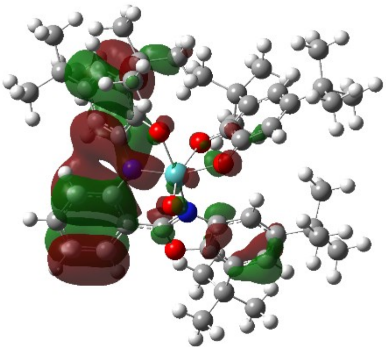
**HOMO-8** 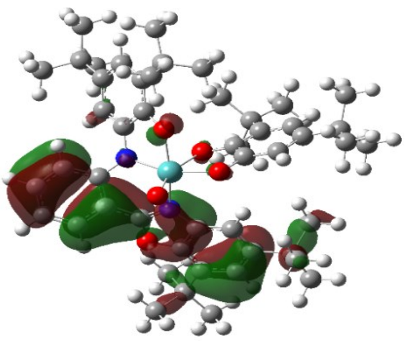	**LUMO** 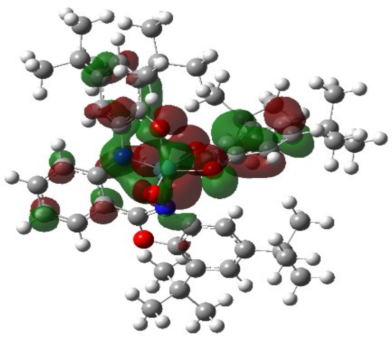

**LUMO+1** 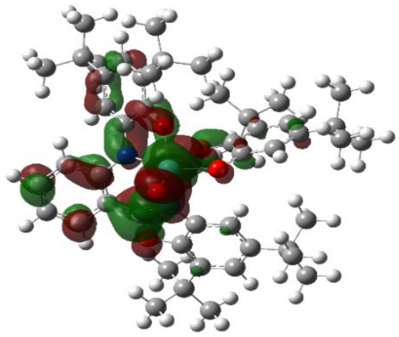	**LUMO+2** 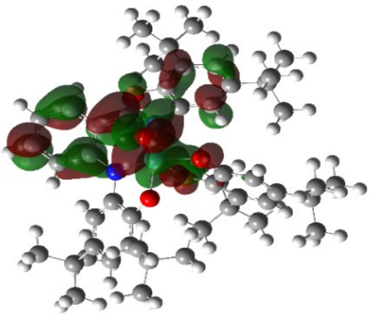
**HOMO+3** 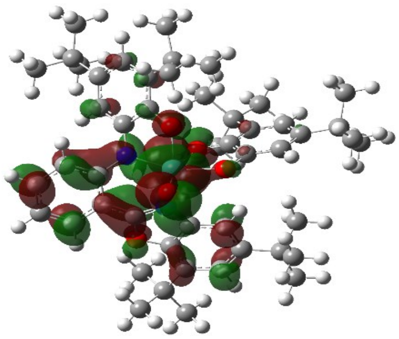	**HOMO+4** 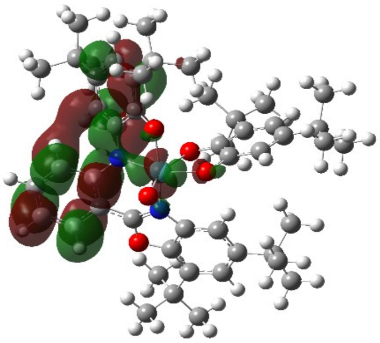

Table S8 The result of blank test for oxidation of phenyl sulfide with some catalytic systems

Entry	Catalytic system	Time (h)	Conversion (%)
1	No catalyst/H_2O_2	8	-
1	H_2L^{BIS}/H_2O_2	8	31
2	$MoO_2(acac)_2/H_2O_2$	8	35
3	$MoO_2(acac)_2/$ H_2L^{BIS}	8	20
4	$MoO_2(acac)_2/$ $H_2L^{BIS}/$ H_2O_2	8	39
5	$MoOL^{BIS}L^{SQ}$/Sulfide	2	-

Reactions condition: Catalyst (2 mol%), H_2O_2 (3 equiv.), acetone as solvent (2 mL), R T.

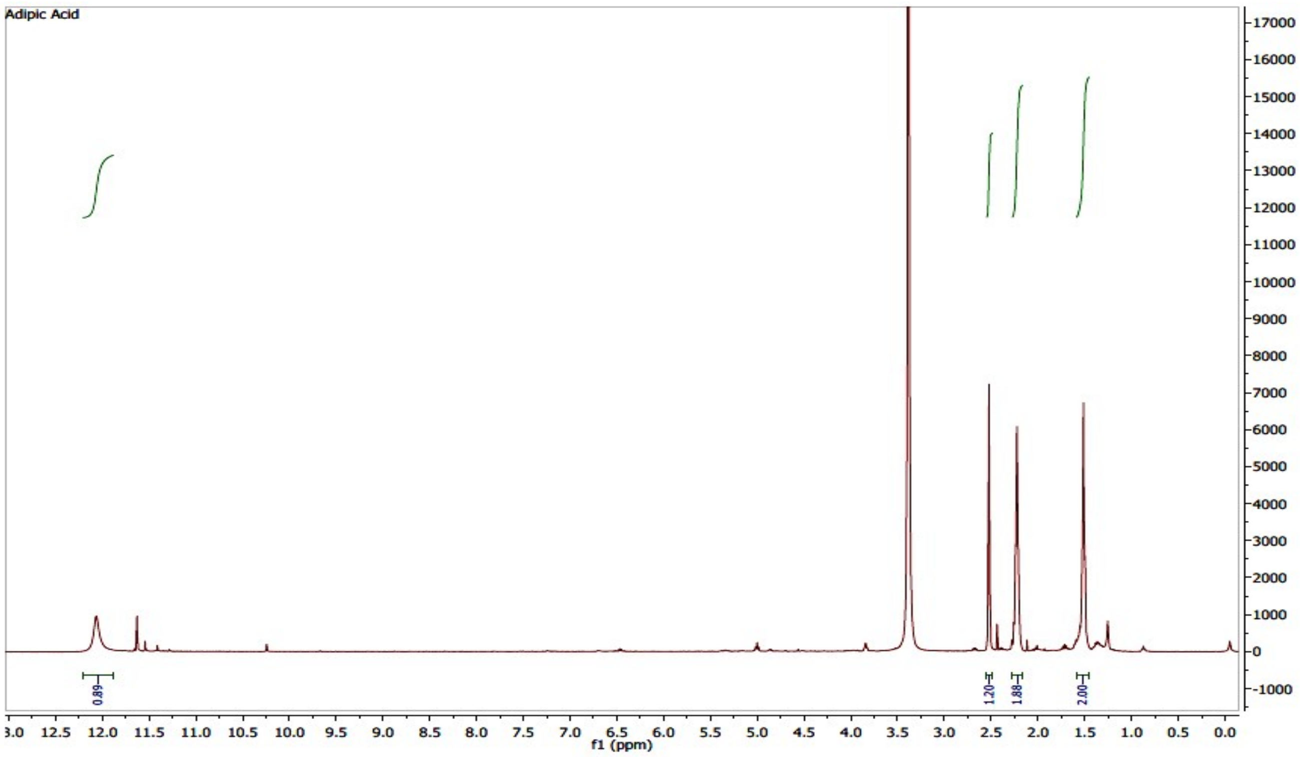

Figure S6 ^1H NMR spectrum of adipic acid

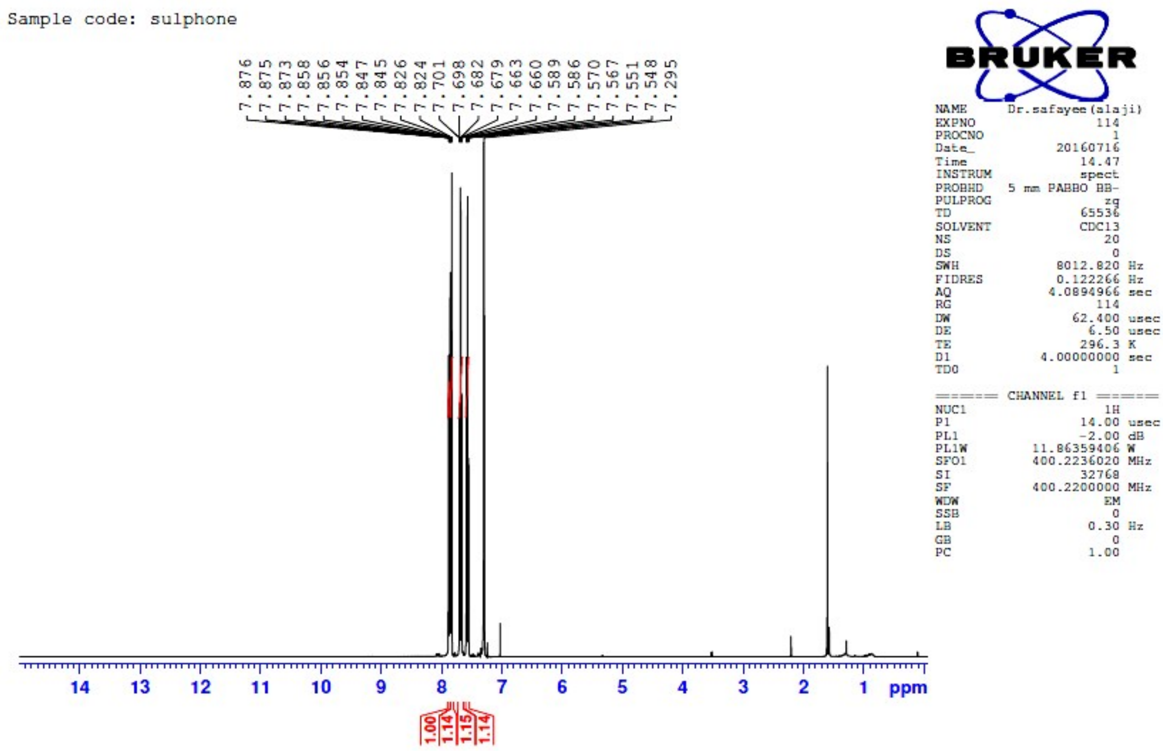

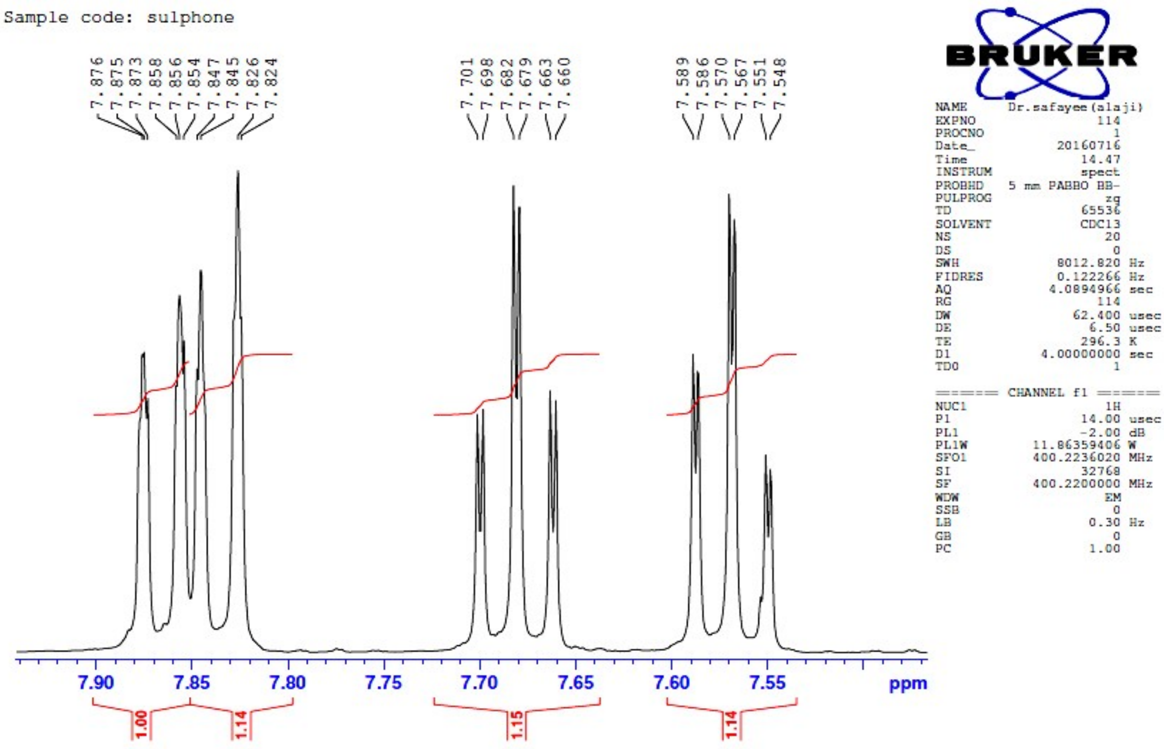

Figure S7 ^{1}H NMR spectrum of dibenzothiophene sulfone

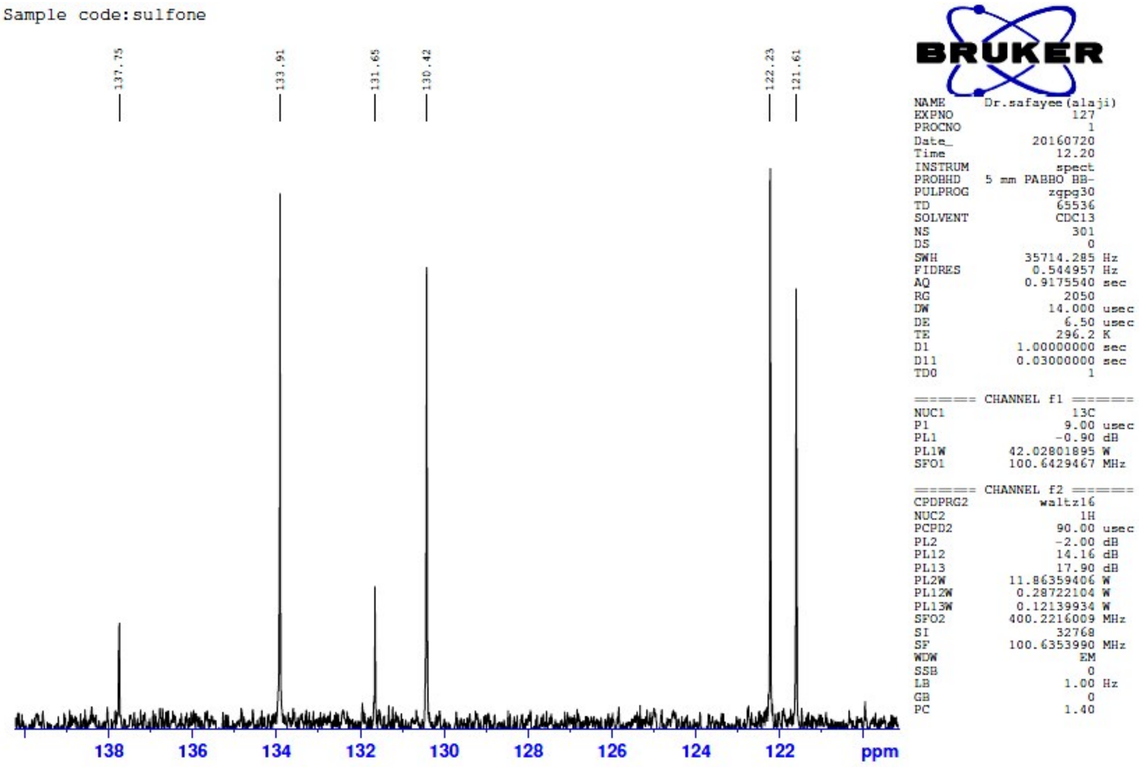

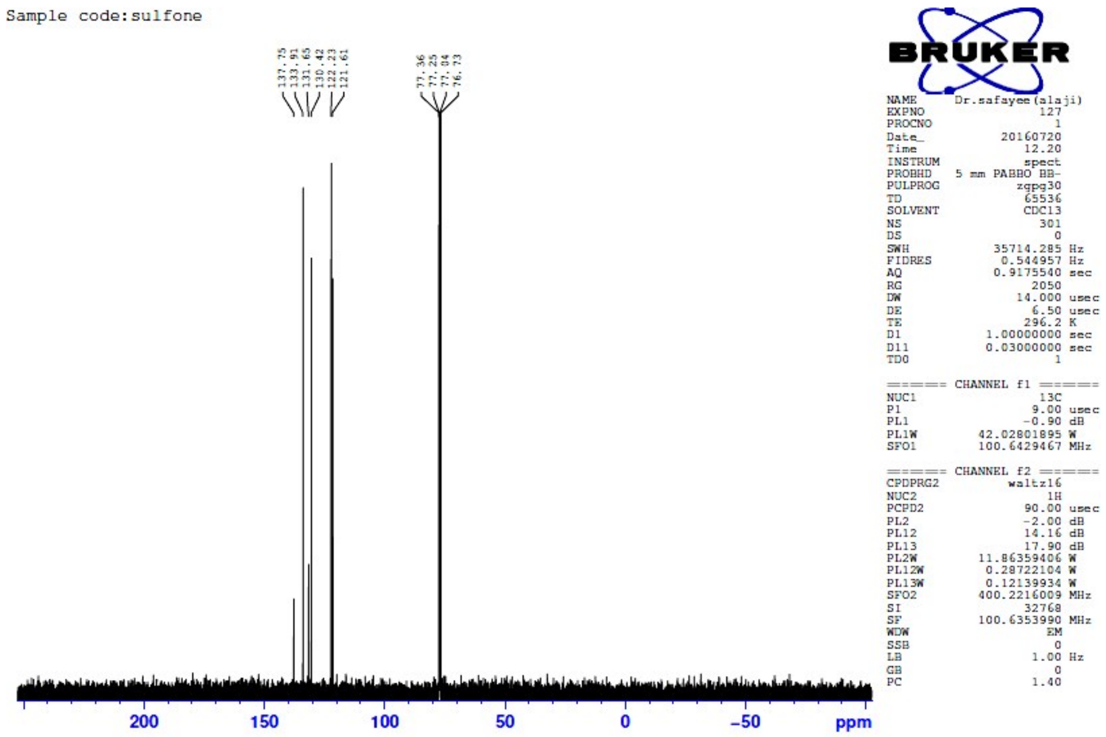

Figure S8 ^{13}C NMR spectrum of dibenzothiophene sulfone
